# Two fungal flavonoid-specific glucosidases/rutinosidases for rutin hydrolysis and rutinoside synthesis under homogeneous and heterogeneous reaction conditions

**DOI:** 10.1186/s13568-021-01298-2

**Published:** 2021-10-18

**Authors:** Michael Kotik, Hana Javůrková, Katerina Brodsky, Helena Pelantová

**Affiliations:** 1 Institute of Microbiology of the Czech Academy of Sciences, Vídeňská 1083, 14220 Prague 4, Czech Republic; 2 Department of Biochemistry and Microbiology, University of Chemistry and Technology Prague, Technická 3, 16628 Prague 6, Czech Republic

**Keywords:** Transglycosylation, Flavonoid glycoside, Solubility, Dimethyl sulfoxide, Process optimization, Enzyme stability

## Abstract

The glycosidases within GH5-23 cleave the glycosidic bond of β-glucosylated or rutinosylated flavonoids. Moreover, by virtue of their transglycosylation activity, glycoconjugates with glucosyl and rutinosyl moieties are accessible. Here we report the biochemical characterization and biotechnological assessment of two heterologously expressed members of GH5-23—*Mc*Glc from *Mucor circinelloides* and *Pc*Glc from *Penicillium chrysogenum*. Both enzymes exhibited the highest hydrolytic activities with quercetin-3-β-*O*-glucopyranoside, whereas lower specificity constants were determined with the rutinosides narcissin, rutin and hesperidin. High stabilities against thermal, ethanol and dimethyl sulfoxide-induced inactivation, a very limited secondary hydrolysis of the formed transglycosylation products, and no detectable product inhibition were additional features appropriate for biotechnological applications. The enzymes were compared in their efficiencies to hydrolyze rutin and to synthesize 2-phenylethyl rutinoside under homogeneous and heterogeneous reaction conditions using high rutin concentrations of 100 and 300 mM. Highest transglycosylation efficiencies were achieved with fully dissolved rutin in reaction mixtures containing 25% dimethyl sulfoxide. Molecular docking and multiple sequence alignments suggest that the hydrophobic environment of aromatic residues within the + 1 subsite of GH5-23 glycosidases is very important for the binding of flavonoid glucosides and rutinosides.

**Supplementary Information:**

The online version contains supplementary material available at 10.1186/s13568-021-01298-2.

## Keypoints

Efficient transglycosylation with dissolved rutin in 25% dimethyl sulfoxide

Transglycosylations in solution are preferable to suspension-based reactions

Advantageous kinetic features of *Mc*Glc and *Pc*Glc linked to their + 1 subsites

## Introduction

The synthesis of both unnatural and naturally occurring glycoconjugates is of high importance in many research fields. Their chemical synthesis can be a difficult and time-consuming undertaking due to the lack of highly efficient regio- and stereoselective reagents (Desmet et al. 2012). This manifests itself in lengthy multi-step reactions, requiring protection and de-protection strategies, which often lead to unsatisfactory yields and the formation of waste products (Sheldon 2016). In this respect, retaining glycosidases with their transglycosylation activities are an alternative to chemical methods for the synthesis of tailored carbohydrates (Rather and Mishra 2013; Slámová et al. 2018).

Glycosylation of small molecules, *e.g.*, quercetin, is a common strategy of nature to modify the physicochemical and biochemical properties of these compounds (Desmet et al. 2012). In order to explore the biological activities and interactions of natural and novel glycosylated compounds, their efficient synthesis is highly important for the development of probes, standards and therapeutics.

**Fig. 1:**
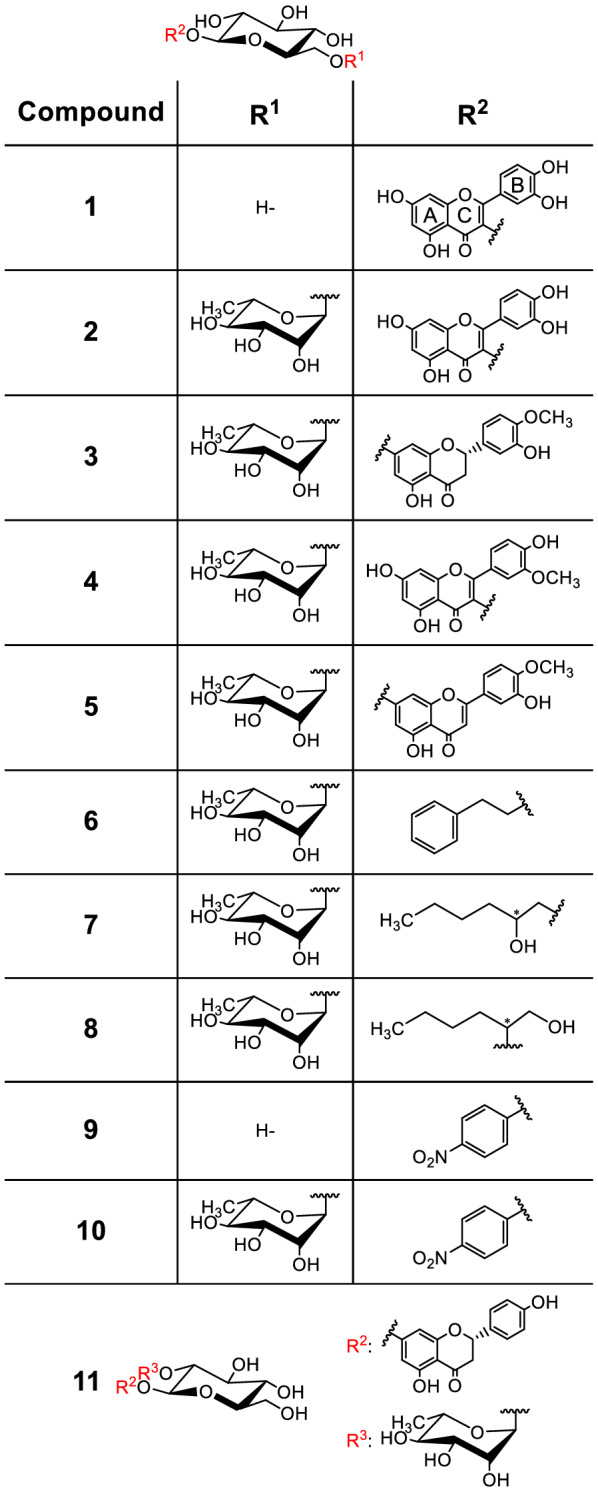
Chemical structures of selected transglycosylation products and flavonoid glycosides tested as substrates. **1**: isoquercitrin (quercetin 3-*O*-β-d-glucopyranoside); **2**: rutin (quercetin 3-*O*-rutinoside); **3**: hesperidin (hesperetin 7-*O*-rutinoside); **4**: narcissin (isorhamnetin 3-*O*-rutinoside); **5**: diosmin (diosmetin 7-*O*-rutinoside); **6**: 2-phenylethyl rutinoside; **7**: hexane-2-ol-1-yl rutinoside; **8**: hexane-1-ol-2-yl rutinoside; **9**: *p*-nitrophenyl β-d-glucoside; **10**: *p*-nitrophenyl rutinoside; **11**: naringin (naringenin 7-*O*-neohesperidoside)

The members of the glycoside hydrolase family 5–23 (GH5-23) belong to the diverse group of diglycosidases, which accept diglycosides such as **2**–**5** as substrates (Fig. 1) (Koseki et al. 2018; Baglioni et al. 2021). So far, two out of six characterized GH5-23 enzymes have been shown to catalyze the hydrolysis of both flavonoid glucosides (*e.g*., **1**) and flavonoid rutinosides (*e.g*., **2**) (Pachl et al. 2020; Makabe et al. 2021). Hence, these broad-specificity enzymes can be classified as β-glucosidases or β-rutinosidases (6-*O*-α-l-rhamnopyranosyl-β-d-glucopyranosidases) depending on their substrate preference. The potential applications of these enzymes are twofold. Their hydrolytic activities can be used for the production of rutinose (6-*O*-α-l-rhamnosyl-d-glucose) and the aglycones quercetin or hesperetin from **2** or **3**, respectively. The hydrolysis products have applications as nutraceuticals and dietary supplements and potentially also in cosmetics (Kiso et al. 2015; Testai and Calderone 2017; Zhang et al. 2018). The synthetic or transglycosylation activities of these enzymes can be used for the synthesis of many novel rutinosylated or glucosylated compounds, including glucosyl and rutinosyl azide (Katayama et al. 2013; Šimčíková et al. 2015; Mazzaferro et al. 2019; Brodsky et al. 2020; Karnišová Potocká et al. 2021; Kotik et al. 2021).

Substantial amounts of rutinosylated flavonoids such as **2** or **3** occur in various plants and their fruits or seedpods (Lucci and Mazafera 2009; O'Shea et al. 2012; Williamson 2017), which are convenient natural sources for the commercialization of **2** and **3** as nutritional supplements (Gullon et al. 2017). Thus, **2** and **3** may be considered readily available and inexpensive glycosyl donors for enzymatic glycosylation reactions. However, their very low water solubility is problematic and poses a challenge for efficient enzymatic hydrolyses/transglycosylations.

In this work, we biochemically characterized two recombinant members of GH5-23, *Mc*Glc from *Mucor circinelloides* and *Pc*Glc from *Penicillium chrysogenum*, with regard to their use as biocatalysts in hydrolytic and transglycosylation reactions. Both enzymes were shown to be flavonoid-specific glucanases/rutinosidases with their highest activities towards isoquercitrin. The enzymes exhibited good activities with flavonoid rutinosides, allowing their use in hydrolyses and transglycosylations under homogeneous and heterogeneous reaction conditions with **2** as a substrate and glycosyl donor. We observed complete hydrolysis of **2** in suspensions with **2** at concentrations of 0.3 M. Regarding transglycosylations, we demonstrated that reaction conditions of fully dissolved **2** in the presence of 25% dimethyl sulfoxide (DMSO) were superior to heterogeneous reaction conditions in terms of yield and process performance data. The present study unveiled the potential of DMSO-tolerant *Mc*Glc and *Pc*Glc for efficient synthetic applications in DMSO-based reaction mixtures with scarcely water-soluble **2** as an inexpensive, readily available and natural glycosyl donor.

## Materials and methods

### Chemicals

Rutin (quercetin 3-*O*-rutinoside) (**2**) was purchased from Alchimica s.r.o. (Czech Republic). Hesperidin (hesperetin 7-*O*-rutinoside) (**3**), narcissin (isorhamnetin 3-*O*-rutinoside) (**4**), diosmin (diosmetin 7-*O*-rutinoside) (**5**), and *p*-nitrophenyl β-d-glucoside (**7**) were purchased from Sigma-Aldrich. Isoquercitrin (quercetin 3-*O*-β-d-glucopyranoside) (**1**) and *p*-nitrophenyl rutinoside (**8**) were prepared as previously described (Weignerová et al. 2012; Šimčíková et al. 2015).

### Expression and purification of recombinant McGlc and PcGlc

During cultivation of the recombinant *Pichia pastoris* KM71H strains, *Mc*Glc and *Pc*Glc were secreted to the culture medium (Kotik et al. 2021). *Pc*Glc was purified by cation exchange chromatography as described in Kotik et al. (2021). *Mc*Glc was purified using a Ni Sepharose column (5-ml HisTrap HP, ÄKTA 900 FPLC system; GE Healthcare Life Sciences) equilibrated with binding buffer (20 mM phosphate, 500 mM NaCl, 20 mM imidazole, pH 7.4). The enzyme was eluted with a linear gradient of imidazole (500 mM final concentration) using a flow rate of 3 ml min^−1^ and a gradient time of 40 min. The purified proteins were concentrated using Amicon Ultra-0.5 and Ultra-15 centrifugal filters (10 kDa cutoff; Merck KGaA, Germany). Protein concentrations were determined using a Qubit protein assay kit with a Qubit fluorometer (Life Technologies, USA). *Mc*Glc and *Pc*Glc were analyzed for the presence of posttranslationally attached carbohydrates using a kit for glycoprotein staining (Pierce – ThermoFisher Scientific).

### Enzymatic reactions

All activity determinations were performed with purified enzymes. Hydrolytic rates with substrates **1**–**5** were determined in 50 mM citrate buffers in the presence of 10% (v/v) DMSO. The reaction mixtures were incubated for 10–15 min, after which the enzyme was inactivated at 95 °C for 30 min. One unit (U) of *Mc*Glc or *Pc*Glc-based activity was defined as the amount of enzyme needed for the release of 1 µmol of quercetin per min using **2** as a substrate (2.0 mM) at optimal pH and 48° C. Compounds **1**–**6** and the released aglycones were quantified by HPLC using the appropriate calibration curves and dilutions in DMSO (see Additional file 1: Table S1 for the retention times and the detection wavelengths). HPLC was performed using a Prominence LC-20AB system (Shimadzu, Japan) at 30 °C as described by Šimčíková et al. (2015) with slightly modified gradients: 0–3 min, 7–30% B; 3–5 min, 30% B; 5–7 min, 30–7% B; 7–7.5 min, 7% B. Mobile phase A: CH_3_CN/H_2_O/HCO_2_H (5:95:0.1; v/v/v); mobile phase B: CH_3_CN/H_2_O/HCO_2_H (80:20:0.1; v/v/v). The reactions were also monitored by thin-layer chromatography as described by Mazzaferro et al. (2019) and Kotik et al. (2021). For transglycosylations, a 200-µL reaction in the absence or presence of DMSO (10 or 25%) was initiated at 35 °C in McIlvaine buffer (pH 4.5 for *Mc*Glc or pH 5.0 for *Pc*Glc) for each data point. Preparative scale conversions with **2** (5.0 mmol) as the glycosyl donor and 2-phenylethanol or 1,2-hexanediol (25 mmol) as the acceptor were performed overnight at 30 °C on a 15-mL scale with purified *Mc*Glc or *Pc*Glc (2.3 mg). The rutinosides **6** and **7**/**8** were purified as described in Brodsky et al. (2020) using a Biogel P2 Fine column (Bio-Rad).

### Effect of pH, temperature and DMSO on enzymatic activities

The pH dependence of the hydrolytic activities of *Mc*Glc and *Pc*Glc was determined in 50 mM citrate buffers, incubating the samples without substrate at 35 °C for 30 min, and then at 48 °C for 15 min in the presence of 2.7 mM **2** and 10% (v/v) DMSO. The temperature optima for the hydrolysis of **2** were determined at pH 3.5 for *Mc*Glc and pH 5.0 for *Pc*Glc in the presence of 10% (v/v) DMSO. The thermal stabilities were assessed by determining the residual hydrolytic activities after a 10-min incubation at a given temperature at pH 4.0 or 5.0 for *Mc*Glc or *Pc*Glc, respectively. The effect of DMSO or ethanol on the hydrolytic activities of both enzymes was determined by varying the co-solvent concentrations in the range of 0–70% (v/v), respectively.

### Structure determination

NMR spectra were recorded on a Bruker Avance III 400 MHz (399.87 MHz for ^1^H, 100.55 MHz for ^13^C, D_2_O, 30 °C, **6**) and a Bruker Avance III 700 MHz spectrometer (700.13 MHz for ^1^H, 176.05 MHz for ^13^C, D_2_O and MeOD (9:1 [v/v]), 30 °C, **7**/**8**). The residual signal of water was used as an internal standard (δ_H_ 4.732 ppm); carbon chemical shifts were referenced to acetone (δ_C_ 30.50 ppm). The following NMR experiments were performed using the manufacturer’s software: COSY, ^1^H-^13^C HSQC, HSQC-TOCSY, 1D-TOCSY and ^1^H-^13^C HMBC. Mass spectra were recorded with a Shimadzu Prominence LC analytical system (Shimadzu, Japan) as described previously (Kotik et al. 2021).

### Bioinformatics

Multiple sequence alignments were performed using CLUSTALW (Thompson et al. 1994). Homology models of *Mc*Glc and *Pc*Glc were built using the automated modeling server SWISS-MODEL (Waterhouse et al. 2018) with the X-ray structure data of *An*Rut (rutinosidase from *Aspergillus niger*; Pachl et al. 2020) as a template (Protein Data Bank accession code 6I1A). Molecular docking was conducted using the program VINA (Trott and Olson 2010), which was implemented in the software YASARA using default settings (Krieger and Vriend 2014).

## Results

### Conserved aromatic residues in + 1 subsite

**Fig. 2:**
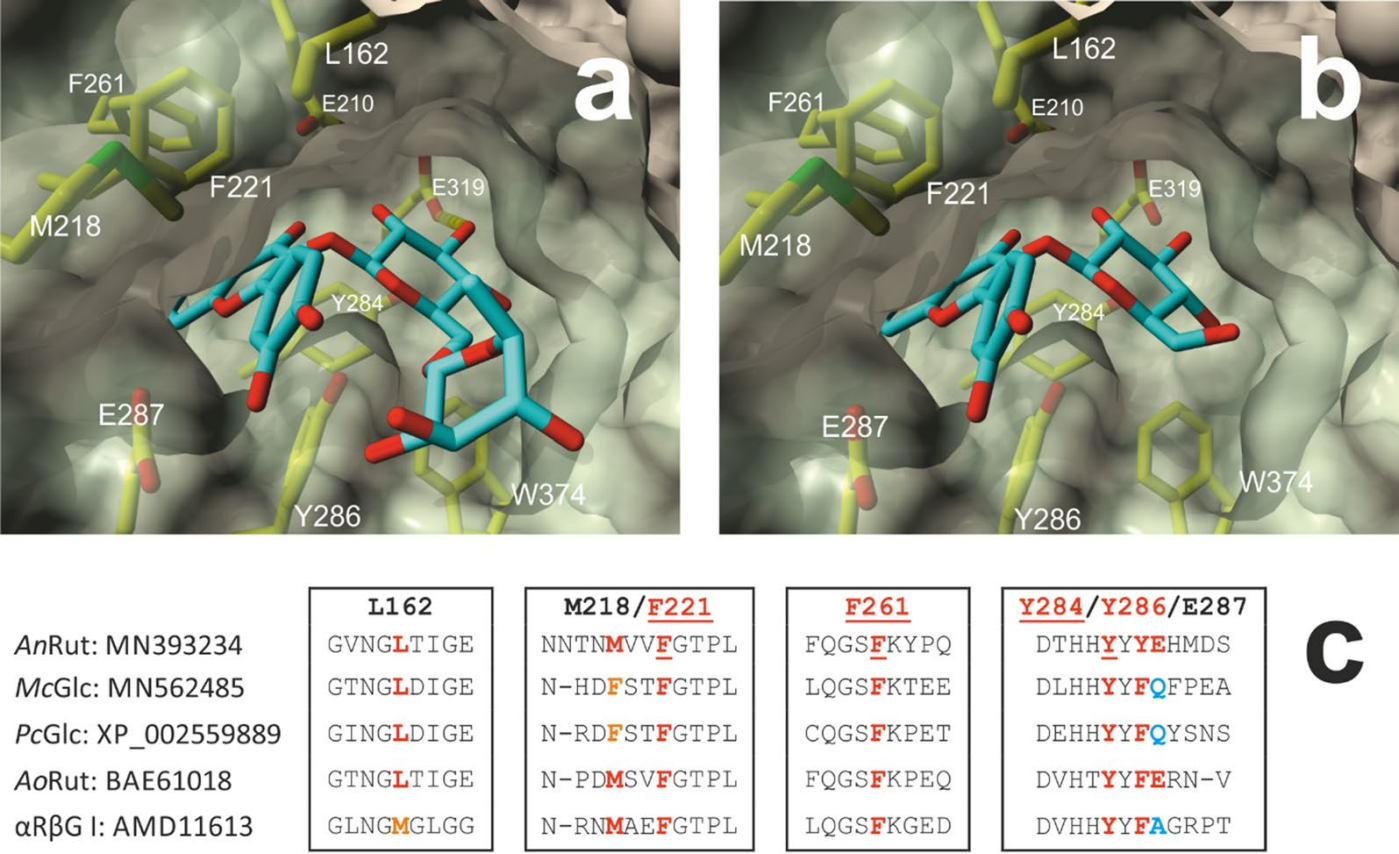
View of the main entrance to the active site of *An*Rut with bound substrate **1** or **2** and multiple sequence alignment of GH5-23 member enzymes. **a** Molecular model of **2** docked to the active site with a free energy of binding of –10.2 kcal mol^–1^. **b** Molecular model of docked **1** with a free energy of binding of –9.3 kcal mol^–1^. Residues that interact with the bound substrates are labeled and shown in stick representation. These include in particular the aromatic sidechains of F221, F261, Y284 and Y286, which interact with the aglycones via many hydrophobic and π–π interactions (Additional file 1: Tables S2–S5). In addition, hydrophobic interactions between the aglycones and the sidechains of L162, M218 and E287 were also found. The catalytic nucleophile E319 and the acid/base catalyst E210 are shown as well. The most favorable binding poses of **1** and **2** are shown. The double ring moiety of quercetin (rings A and C; see Fig. 1) is buried in the side tunnel and only incompletely visible. The above-mentioned residues were set flexible during the simulation. The *An*Rut-based residues are covered with a partially transparent molecular protein surface. **c** Multiple sequence alignment of GH5-23 member enzymes. Only segments around residues that—according to the molecular docking results—are involved in hydrophobic and π-π interactions between the quercetin moiety of **1** or **2** and the + 1 subsite of *An*Rut are shown. The aromatic residues F221, F261 and Y284 are fully conserved. The residues at the positions 162, 218 and 286 are partially conserved. The numbering refers to the sequence of *An*Rut (MN393234) (Pachl et al. 2020). The following sequences were aligned: MN562485 (*Mc*Glc) and XP_002559889 (*Pc*Glc) (Kotik et al. 2021), BAE61018 (*Ao*Rut; Makabe et al. 2021), AMD11613 (αRβG I; Weiz et al. 2019)

Sequence identities of 56 and 60% were determined between *An*Rut, whose X-ray structure had been determined (Pachl et al. 2020), and *Mc*Glc and *Pc*Glc, respectively; this allowed automated structure model building with high quality estimates (Benkert et al. 2011). The superposition of the *An*Rut structure and the generated models of *Mc*Glc and *Pc*Glc revealed a very high degree of structural similarity of the backbone chains of the three enzymes (Additional file 1: Fig. S1). Furthermore, a sequence alignment of *An*Rut, *Mc*Glc and *Pc*Glc enabled us to predict the two key catalytic residues – the acid/base catalyst and the catalytic nucleophile (Additional file 1: Fig. S1). Molecular docking of substrate **1** or **2** to the active site of *An*Rut resulted in very similar binding poses with the quercetin moieties being clamped by four aromatic side chains, comprising Phe and Tyr, located in the + 1 subsite (Fig. 2). The analysis of the interactions between quercetin and the side chains in the + 1 subsite indicated that predominantly hydrophobic and π-π interactions were responsible for the binding of the aglycone to the + 1 subsite (Additional file 1: Tables S2–S5; Fig. S2). Interestingly, these four aromatic side chains in *An*Rut were found conserved in a multiple sequence alignment with other GH5-23 member enzymes, including *Mc*Glc and *Pc*Glc (Fig. 2).

### Production of recombinant enzymes

Typical volumetric productivities of 4-d long cultivations in BMM medium using baffled flasks were 2.2–2.4 U mL^−1^ d^−1^. Typical purification yields for *Mc*Glc and *Pc*Glc were 30–45 and ~ 50%, respectively. During purification, the specific activity of *Mc*Glc for the hydrolysis of **2** increased from 26 to 30 µmol min^–1^ mg^–1^, the corresponding data for *Pc*Glc were 45 and 55 µmol min^–1^ mg^–1^.

### Biochemical properties

**Fig. 3:**
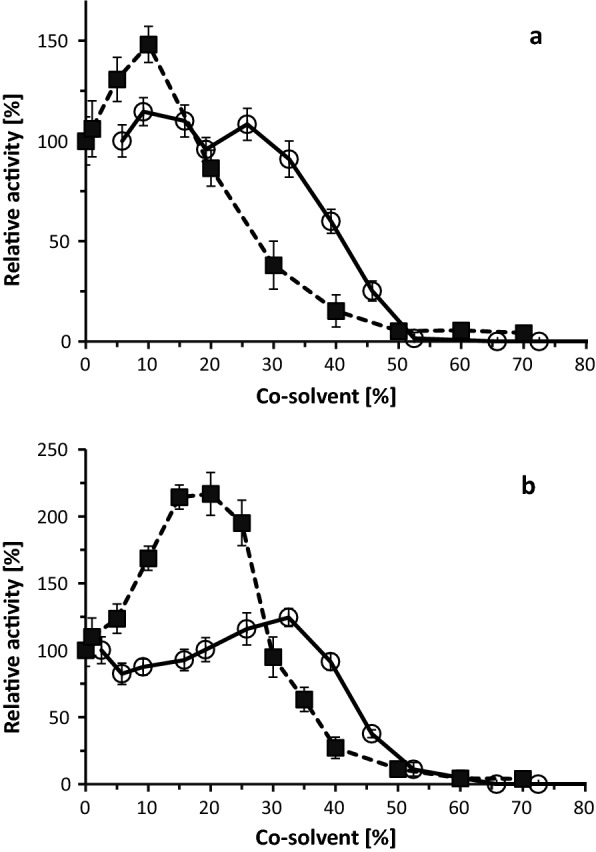
Influence of ethanol and DMSO on the initial hydrolysis rate of **2**. **a** *Mc*Glc in 50 mM citrate buffer (pH 3.5) at 48 °C; **b** *Pc*Glc in 50 mM citrate buffer (pH 5.0) at 48 °C. The measurements were performed in triplicate in the presence of different co-solvent concentrations (%, v/v): 2 mM of **2** with varying concentrations of ethanol (■); DMSO (○)

The presence of glycan chains attached to heterologously produced and purified *Mc*Glc and *Pc*Glc was clearly detected using a sugar-specific staining method based on periodic acid and the Schiff reagent (Additional file 1: Fig. S3). The purified enzymes were further characterized in terms of their pH optima and stabilities. The optimal pH of *Mc*Glc for the hydrolysis of **2** was determined between 3.5 and 4.0. *Pc*Glc exhibited a shift in the activity-pH relationship towards less acidic conditions; the optimal pH was determined between 5.0–5.5 (Additional file 1: Fig. S4). The maximal hydrolytic activities were determined at 55 and 60 °C for *Mc*Glc and *Pc*Glc at their optimal pH values, respectively (Additional file 1: Fig. S5). After 10 min of incubation in the absence of substrate, the enzymes retained > 90% of their initial activities in the temperature range of 20–55 °C (Additional file 1: Fig. S6). As DMSO can be used for solubilizing flavonoids in reaction mixtures, we determined its influence on the enzymatic activities. It turned out that both enzymes showed a high degree of tolerance towards this solubilizing agent with activity losses being observed only above 30–35% (Fig. 3). Low concentrations of ethanol resulted in higher initial velocities of hydrolysis, whereas concentrations of > 20 and > 30% led to a decrease in activity for *Mc*Glc and *Pc*Glc, respectively (Fig. 3).

### Hydrolysis in solution – substrate specificities

The substrate specificities of *Mc*Glc and *Pc*Glc were determined with a range of potential substrates: **1**, which is a flavonol glucoside, and four flavonoid rutinosides (**2**–**5**) (Fig. 1). With the exception of **5** (Additional file 1: Fig. S7), classical Michaelis–Menten kinetics was observed with both enzymes for compounds **1**–**4** (Additional file 1: Fig. S8–S11). Regarding the specificity constants *k*_cat_/*K*_M_ (Table 1), the best substrate by far was glucoside **1**. The following substrate order – from best to worst – was determined for both *Mc*Glc and *Pc*Glc: **1** > **4** > **2** > **3**. Neither *Mc*Glc nor *Pc*Glc exhibited activities towards compound **11**.

**Table 1:** Kinetic constants for the hydrolysis of compounds **1**–**4** using *Mc*Glc or *Pc*Glc as a catalyst

Compound	Kinetic constants	*Mc*Glc	*Pc*Glc
**1**	*k*_cat_ [s^–1^]	53.9 ± 1.9	30.3 ± 1.5
	*K*_M_ [mM]	0.04 ± 0.01	0.03 ± 0.006
	*k*_cat_/*K*_M_ [s^–1^ mM^–1^]	1348	1010
	*k*_cat_ [s^–1^]	22.7 ± 0.5	39.3 ± 1.2
**2**	*K*_M_ [mM]	0.12 ± 0.03	0.21 ± 0.06
	*k*_cat_/*K*_M_ [s^–1^ mM^–1^]	189	187
**3**	*k*_cat_ [s^–1^]	23.0 ± 1.7	9.3 ± 0.8
	*K*_M_ [mM]	4.8 ± 0.1	0.38 ± 0.09
	*k*_cat_/*K*_M_ [s^–1^ mM^–1^]	4.8	24.5
**4**	*k*_cat_ [s^–1^]	69.1 ± 6.0	120 ± 13
	*K*_M_ [mM]	0.21 ± 0.09	0.29 ± 0.1
	*k*_cat_/*K*_M_ [s^–1^ mM^–1^]	257	414

The specific hydrolytic activities for the artificial substrate **9** were found markedly lower by a factor of ~ 350 compared with the corresponding data of **1** (Additional file 1: Table S6). Much lower activities were also determined for compound **10** in comparison with **2** for both *Mc*Glc and *Pc*Glc. Moreover, the hydrolysis of **2** was virtually unaffected by the presence of β-d-glucose, α-l-rhamnose and rutinose (Additional file 1: Table S6).

It is well known that secondary hydrolysis of reaction products results in lower product yields. Incubating *Mc*Glc or *Pc*Glc with selected transglycosylation products revealed that virtually no hydrolysis was detectable by thin-layer chromatography (Additional file 1: Fig. S12). This outcome appears to be in line with the absence of a detectable inhibition of hydrolytic reactions with substrate **2** by moderate concentrations (< 12 mM) of transglycosylation product **6** (Additional file 1: Fig. S13).

### Transglycosylation reactions in solution

**Fig. 4:**
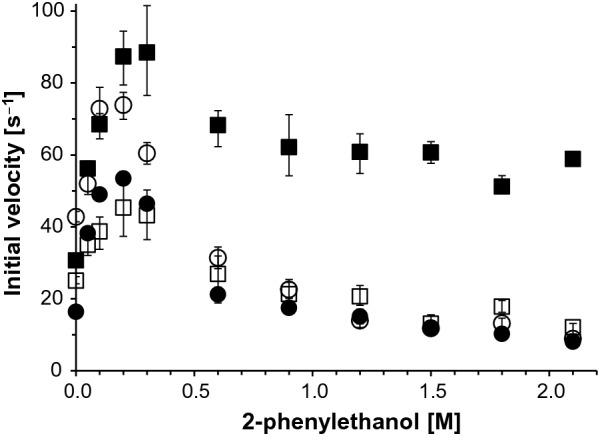
Initial velocities of transglycosylation reactions with *Mc*Glc and *Pc*Glc as catalysts in the presence of **1** or **2** and various concentrations of 2-phenylethanol. The total activities (hydrolysis plus transglycosylation) are shown for **1** (*Mc*Glc, ○; *Pc*Glc, □) or **2** (*Mc*Glc, ●; *Pc*Glc, ■) as a substrate/glycosyl donor. Reaction conditions: 37 °C, 10% DMSO, 1 mM of compound **1** or 2 mM of **2**, 0.25–1.0 µg mL^−1^ of enzyme, 50 mM citrate buffer at pH 4.0 (*Mc*Glc) or pH 5.0 (*Pc*Glc)

In an attempt to compare the transglycosylation capabilities of *Mc*Glc and *Pc*Glc, various acceptors were tested in the presence of solubilized glycosyl donor **2** and 10% (v/v) DMSO (Table 2). We did not detect any differences in acceptor preference between *Mc*Glc and *Pc*Glc, which underlines the high structural similarity of the enzymes. Interestingly, methanol, ethanol, pentan-2-ol, catechol and geraniol were rejected as acceptors, in contrast to *An*Rut. In an additional experiment, the total enzymatic activities in transglycosylations with 2-phenylethanol as a model acceptor and **1** or **2** as a glycosyl donor were compared between *Mc*Glc and *Pc*Glc-mediated reactions (Fig. 4). For both enzymes and glycosyl donors, we observed the highest total activities at an acceptor concentration of 0.2–0.3 M. Concentrations exceeding ~ 0.3 M led to a decrease in overall activity, reaching different activity levels for *Pc*Glc in the presence of **2** or **1**.

**Table 2:** Acceptor specificities for transglycosylation reactions with **2** as rutinosyl donor using *Mc*Glc or *Pc*Glc^a^ as a catalyst—comparison with *An*Rut^b^

Acceptor	*Mc*Glc	*Pc*Glc	*An*Rut
Methanol	−^c^	−	+
Ethanol	−	−	+
*n*-Propanol	+ ^d^	+	+
*n*-Butanol	+	+	+
Pentan-1-ol	+	+	n.d
Pentan-2-ol	−	−	+
Hexan-1-ol	+	+	n.d
Catechol	−	−	+
Geraniol	−	−	+
2-Phenylethanol	+	+	+
2-Azidoethanol	+	+	+
1,2-Hexanediol	+	+	n.d
3-Allyloxy-1,2-propanediol	+	+	n.d
Sodium azide^e^	+	+	+

*n.d.* not determined

^a^Based on results obtained by thin-layer chromatography. Reaction conditions: 47 °C, 1.8 mM **2**, 0.12 µg mL^–1^ enzyme, 50 mM citrate buffer at pH 4.0 (*Mc*Glc) or pH 5.0 (*Pc*Glc), 10% (v/v) DMSO

^b^See Šimčíková et al. (2015) and Brodsky et al. (2020); including unpublished results

^c^No transglycosylation product detected

^d^Transglycosylation product detected

^e^SeeKotik et al. (2021)

The transglycosylation performance of *Mc*Glc and *Pc*Glc was further compared using 2-phenylethanol as an acceptor with 25% (v/v) DMSO in the reaction mixture. The reaction conditions ensured full solubilization of glycosyl donor **2** at a concentration of 100 mM. Using an S/E ratio of 67′000 mol mol^–1^, the optimal acceptor concentration for the highest rutinoside production was determined to be 200 and 400 mM for *Mc*Glc and *Pc*Glc, respectively (Additional file 1: Fig. S14). Regarding yield, *Pc*Glc exhibited a slightly better performance with a maximum yield of 49% for synthesized **6** (based on the initial concentration of **2**) in comparison with *Mc*Glc with a maximum yield of 43%. The reaction time courses are shown in Additional file 1: Fig. S15. The *Pc*Glc-based transglycosylation yields were found independent of the S/E ratio in the range of 20′000 to 67′000 mol mol^–1^. A lower transglycosylation yield of 28% for *Mc*Glc in the presence of 400 mM acceptor was improved to 40% by augmenting the enzyme load (*i.e*., reducing the S/E ratio from 67′000 to 20′000 mol mol^–1^) (Additional file 1: Fig. S16). The calculated process parameters for low and high conversions of **2** are listed for the optimized transglycosylation reactions in Table 3.

**Table 3:** Performance of *Mc*Glc and *Pc*Glc in optimized transglycosylation reactions at low and high conversions with dissolved **2** and 2-phenylethanol^a^

	TOF^c^		Productivity^d^		STY^e^	
X^b^	0.2	0.7	0.2	0.7	0.2	0.7
*Mc*Glc	13	6.9	1240	640	86	44
*Pc*Glc	8.9	2.9	901	292	57	19

^a^Using a substrate to enzyme (S/E) ratio of 67,000 mol mol^–1^. Reaction conditions: 35 °C, 25% (v/v) DMSO, 100 mM initial concentration of **2**; 200 or 397 mM of 2-phenylethanol for *Mc*Glc or *Pc*Glc-based reactions, respectively. The quantification of quercetin and compound **6** enabled the determination of the total (*i.e*., hydrolysis and transglycosylation) and the transglycosylation-specific conversion, respectively

^b^X: conversion of glycosyl donor **2**

^c^Turnover frequency: 10^4^ mol of **6**·(mole of catalyst·h)^–1^

^d^Catalyst productivity: g of **6**·(g catalyst·h)^–1^

^e^Space–time yield: g of **6**·(L·h)^–1^

### Hydrolysis in suspensions

The performance of *Mc*Glc and *Pc*Glc was further analyzed for hydrolytic reactions with mostly undissolved **2** at a concentration of 185 g L^–1^ (0.3 M) in the absence of a co-solvent. Although the reaction mixtures were thick suspensions, complete conversions were observed for both enzymes (Additional file 1: Fig. S17). Turnover frequency (TOF) values of ~ 13′000 h^–1^ for the first hour of the 16-h long processes were determined for both *Mc*Glc and *Pc*Glc-based conversions. A summary of the performance metrics of the hydrolytic processes is shown in Table 4. It should be noted that one of the reaction products, quercetin, is virtually insoluble in water, consequently, as the reaction proceeded, the mixture remained in a state of a slurry (Kapešová et al. 2019).

**Table 4:** Performance of *Mc*Glc and *Pc*Glc in hydrolysis reactions at low and high conversions with **2** in suspension^a^

	TOF^c^		Productivity^d^		STY^e^	
X^b^	0.2	0.7	0.2	0.7	0.2	0.7
*Mc*Glc	1.7	0.7	108	45	36	15
*Pc*Glc	1.5	0.6	108	45	36	15

^a^Using an S/E ratio of 40′000 mol mol^–1^; 35 °C; initial concentration of **2**: 300 mM

^b^X: conversion of substrate **2**

^c^Turnover frequency: 10^4^ mol of quercetin·(mole of catalyst·h)^–1^

^d^Catalyst productivity: g of quercetin·(g catalyst·h)^–1^

^e^Space–time yield: g of quercetin·(L·h)^–1^

### Transglycosylations in suspensions

Before we embarked on the optimization of the transglycosylation reactions under heterogeneous conditions with **2** as the glycosyl donor, we performed not optimized reactions using either catalyst and 2-phenylethanol or *rac*-1,2-hexanediol as an acceptor. Two reactions were performed on a 15-ml scale using the combinations *Mc*Glc/2-phenylethanol and *Pc*Glc/1,2-hexanediol (Additional file 1: Fig. S18). The reaction products were isolated and subsequently characterized by mass and NMR spectrometry (compound **6**: Additional file 1: Table S7 and Fig. S19–S21; compounds **7**/**8**: Additional file 1: Tables S8 and S9, Fig. S22–S24). The acquired data were congruous with the expected structures of **6** and **7**/**8**. The configuration at the anomeric centers was determined to be *beta* as inferred from the H-1/H-2 coupling constants (*J*_H1,H2_ = 7.9–8.0 Hz; Additional file 1: Tables S7–S9). The connection between the monosaccharide unit and the aglycone was confirmed using corresponding HMBC correlations. In the presence of *rac*-1,2-hexanediol two pairs of epimers were produced with the glycosidic bond linked either to the methylene bridge (compound **7**) or to the methanetriyl group (compound **8**). Compounds **7**/**8** were isolated as a mixture of two pairs of epimers with compound **7** in a 2.3-fold excess over compound **8** as determined by NMR analysis (Additional file 1: Table S8).

Next, we undertook a detailed comparison between the transglycosylation capabilities of *Mc*Glc and *Pc*Glc with 2-phenylethanol as a model acceptor compound. The reaction mixtures contained 185 g L^–1^ (0.3 M) of **2** as a glycosyl donor, forming a slurry. In a first attempt, the enzyme load was tested. It turned out that the maximal transglycosylation yield was independent of the S/E ratio between 15′000 to 55′000 mol mol^–1^ for both enzymes. Next, the correlation between acceptor concentration and rutinoside formation was studied. Using *Mc*Glc as a catalyst, the data indicated a slightly higher relative conversion towards the transglycosylation product **6** at high acceptor concentrations (~ 1.5 M) in comparison with the total conversion (Additional file 1: Fig. S25). In the case of *Pc*Glc, a rather sharp increase of the conversion of **2** with the acceptor concentration was followed by a moderate increase in both total and transglycosylation-linked conversions (Additional file 1: Fig. S26). As a next step, a time course experiment, in which we followed the production of quercetin and the transglycosylation product **6**, was performed with a common S/E ratio of 55′000 mol mol^–1^. Based on the previous experiments, optimal acceptor concentrations of 1.5 and 1.2 M were selected for the *Mc*Glc and *Pc*Glc-mediated reactions, respectively (Additional file 1: Fig. S27). The reaction efficiencies for both processes are summarized in Table 5 for low and high conversions. Both reactions reached a plateau 8 h after the initiation of the reaction. Higher yields of 97 and 25% for quercetin and product **6** were determined for the *Pc*Glc-mediated process, compared with the *Mc*Glc-based reaction with 75 and 20%, respectively.

**Table 5:** Performance of *Mc*Glc and *Pc*Glc in optimized transglycosylation reactions at low and high conversions with **2** in suspension in the presence of 2-phenylethanol^a^

	TOF^c^		Productivity^d^		STY^e^	
X^b^	0.2	0.7	0.2	0.7	0.2	0.7
*Mc*Glc	2.1 (6.1)	0.14 (0.6)	174 (353)	11 (32)	50 (101)	3.2 (9)
*Pc*Glc	1.2 (3.6)	0.51 (1.9)	135 (284)	57 (149)	29 (61)	12 (32)

^a^Using an S/E ratio of 55′000 mol mol^–1^; 35 °C; initial concentration of **2**: 300 mM. The quantification of quercetin and compound **6** enabled the determination of the total (*i.e*., hydrolysis and transglycosylation) and the transglycosylation-specific conversion, respectively. The data in parentheses were calculated based on quercetin as the reaction product

^b^X: conversion of glycosyl donor **2**

^c^Turnover frequency: 10^4^ mol of **6** or quercetin·(mole of catalyst·h)^–1^

^d^Catalyst productivity: g of **6** or quercetin·(g catalyst·h)^–1^

^e^Space–time yield: g of **6** or quercetin·(L·h)^–1^

## Discussion

### Substrate specificities

As part of the assessment of *Mc*Glc and *Pc*Glc for biotechnological applications, which included two different enzyme purification strategies, we first determined the substrate specificities of these enzymes. From these data we can conclude that—(*1*) small structural variations in the aglycone have a profound influence on the enzyme activity (see **2** *versus* **4** or** 3** *versus* **5**), (*2*) the specificity of *Mc*Glc and *Pc*Glc towards the type of linkage between the two glycone units is very high (1 → 6 *versus* 1 → 2 glycosidic linkage; see **3** *versus* **11**), (*3*) *Mc*Glc and *Pc*Glc appear to prefer flavonoid rutinosides with 3-*O*-linkages over rutinosides with 7-*O*-linkages (see **4** *versus* **5** and **2** *versus* **3**), and (*4*) the presence of a terminal rhamnosyl residue as in compound **2** leads to a lower *k*_cat_ value and a higher *K*_M_ value compared to compound **1**, which lacks the rhamnosyl residue but contains the same aglycone moiety. The presence of a double bond in the heterocyclic ring of **5** (compared to **3**) had a striking effect on the steady-state kinetics of both enzymes; they exhibited an extreme level of what appeared to be substrate inhibition (Additional file 1: Fig. S7). Both *Mc*Glc and *Pc*Glc were shown to be most active with the flavonoid glucoside **1**, as found for *An*Rut (Pachl et al. 2020). High activity towards **1** was also determined for the rutinosidase from *Aspergillus oryzae* (Makabe et al. 2021).

### Interactions between aglycone and + 1 subsite

Based on the above-mentioned data we can conclude that the hydrophobic and π-π interactions between the aromatic side chains in the + 1 subsite of GH5-23 glycosidases and the aglycone of the substrate/glycosyl donor are highly important for binding and fast conversions. The conclusion is supported by: (*i*) the molecular docking results of the in silico binding of **1** and **2** to the *An*Rut active site together with the multiple sequence alignment of GH5-23 glycosidases and their conserved aromatic residues in the inferred + 1 subsite, (*ii*) the very low hydrolytic activities with the substrates **9** and **10**, (*iii*) the virtual absence of secondary hydrolysis of the transglycosylation products, and (*iv*) the fact that moderate concentrations of **6**, glucose, rhamnose or rutinose did not substantially affect the initial hydrolysis rate of compound **2**. In conclusion, substrates and glycosyl donors that lack the aromatic three-ring aglycone structure of compounds **1**–**4** are not congruous with high conversion rates.

### Transglycosylation in solution

In accordance with the determined *beta* configuration at the anomeric centers of the reaction products **6** and **7**/**8**, we concluded that *Mc*Glc and *Pc*Glc function as retaining glycosidases. The increase in the total activity in the presence of **1** or **2** and intermediate 2-phenylethanol concentrations compared with the activity in the absence of the acceptor may have different causes. The nucleophilicity of the active site nucleophile may get stronger in the presence of co-solvents as a result of less intense water solvation of the nucleophile (see Fig. 3 for a similar effect caused by ethanol; Kudryashova et al. 2003). We may also speculate that the rate-limiting step in the transglycosylation reaction pathway is the hydrolysis of the glycosyl-enzyme intermediate (deglycosylation step) and not its formation. In such a case, the addition of 2-phenylethanol as an acceptor to the reaction mixture would increase the total reaction rate as previously observed with inorganic azide (Additional file 1: Fig. S28; Kotik et al. 2021). This appears to be true for both **1** and **2** as substrates/glycosyl donors. Our conclusions are also based on the well-established data of the serine protease chymotrypsin (Fersht 1985), whose reaction mechanism resembles the mechanism of retaining glycosidases in several aspects, including the presence of a covalent intermediate. Acceptor concentrations exceeding ~ 0.3 M led to a decrease in overall activity, possibly due to adverse conformational effects or inhibition caused by two or more acceptor molecules bound to the active site. Concerning the regioselectivity of *Pc*Glc, 1,2-hexanediol was preferentially rutinosylated at the primary hydroxyl group with a selectivity of ~ 70%. Similar data were reported for 1,2-propanediol with a number of β-glycosidases (van Rantwijk et al. 1999).

### Reactions in solution versus in suspension

Compound **2** has a very low water solubility of 125 mg L^–1^ (Frutos et al. 2019). Hence, co-solvents such as DMSO help solubilize **2** and usually ensure faster conversions if catalyst denaturation can be avoided. Indeed, one of the advantageous features of *Mc*Glc and *Pc*Glc is their high resistance against DMSO and ethanol-induced inactivation in comparison with, *e.g*., *An*Rut with its substantial proneness to activity loss in the presence of these co-solvents (Šimčíková et al. 2015). Therefore, *Mc*Glc and *Pc*Glc offer the possibility to perform transglycosylations with dissolved **2** at a high concentration of 100 mM, which are conditions that led to double yields of **6** and much better productivities and TOF values in comparison with the ‘solid-state’ approach (Tables 3 and 5). A potential drawback of DMSO in the reaction mixture may lie in the interference with product work-up. However, the efficient removal of DMSO is possible using solid-state extraction (Crawford et al. 2018). Lyophilization is another option; due to the low vapor pressure of DMSO, it is probably not the preferred method (Müller et al. 2019). Regarding the TOF numbers, both transglycosylation approaches can be considered potentially economical for high-value products (Behr and Neubert 2012). However, the lower yield of **6** for the ‘solid-state’ process is a considerable disadvantage in comparison with the solution-based reaction. The presence of 2-phenylethanol considerably improved the hydrolysis of **2** under heterogeneous reaction conditions (Tables 4 and 5). It is very likely that this effect has to be attributed to a better solubilization and thus an improved availability of **2** as a result of 2-phenylethanol (1.2–1.5 M) that acts both as an aglycone and co-solvent.

The yields of transglycosylation reactions depend on many factors, including the type of the acceptor, the biocatalyst, the acceptor-to-donor ratio, and the co-solvent used (Zeuner et al. 2019). Typically, several reaction parameters have to be optimized to achieve the highest conversion (Guo et al. 2015; Manas et al. 2018; Mazzaferro et al. 2019). Acceptors compete with water during the deglycosylation step of the two-step displacement mechanism of retaining glycosidases (Bissaro et al. 2015). The outcome of this competition in the attack on the covalent glycosyl-enzyme intermediate with its complex interactions determines the transglycosylation to hydrolysis ratio. Notably, optimizations of transglycosylation reactions have their limits, as shown by the fact that transglycosylation yields of more than 50% have rarely been reported (Desmet et al. 2012; Guo et al. 2015)—with the exception of reactions using transglycosidases (Bissaro et al. 2015).

In this study, we focused on the biotechnological potential of two flavonoid-specific glucosidases/rutinosidases, *Mc*Glc and *Pc*Glc. The biochemical characterization of the two GH5-23 family members revealed enzyme characteristics that are highly advantageous for biotransformation reactions. In particular, significant stabilities against thermal and DMSO-induced inactivation, acceptance of both flavonoid glucosides and rutinosides as substrates, transglycosylation capabilities with a considerable number of acceptors, and a very limited secondary hydrolysis of the formed transglycosylation products should be mentioned. Very similar efficiencies for the hydrolysis of **2** at a concentration of 0.3 M were determined for *Mc*Glc and *Pc*Glc. In terms of yields, *Pc*Glc outperformed *Mc*Glc in optimized transglycosylation reactions for the synthesis of compound **6** with **2** as a glycosyl donor under both homogeneous and heterogeneous reaction conditions. The rutinosylation of 2-phenylethanol in the presence of 25% DMSO was demonstrated to lead to much higher efficiencies and double yields of **6** in comparison with the corresponding transglycosylation reactions performed in ‘solid-state’ mode. Molecular docking and multiple sequence alignments point towards hydrophobic and π-π interactions between the flavonoid aglycone and the aromatic residues in the + 1 subsite as a major driving force for substrate recognition.

## Supplementary Information

**Additional file 1.** Additional figures and tables.

## Data Availability

The authors confirm that the data supporting the findings of this study can be found in the article and its supplementary material.
