# Supplementary material for: Two fungal flavonoid-specific glucosidases/rutinosidases for rutin hydrolysis and rutinoside synthesis under homogeneous and heterogeneous reaction conditions
Source: AMB Express. 2021 Oct 18;11:136. doi: 10.1186/s13568-021-01298-2 (PMC8523606; doi:10.1186/s13568-021-01298-2)
Supplement: Supplementary file 1 — Additional file 1. Additional figures and tables. [file 13568_2021_1298_MOESM1_ESM.docx]

**Supplementary material**

**Two fungal flavonoid-specific glucosidases/rutinosidases for rutin hydrolysis and rutinoside synthesis under homogeneous and heterogeneous reaction conditions**

Michael Kotik^1^, Hana Javůrková^1^, Katerina Brodsky^1,2^, Helena Pelantová^1^

^1^ Institute of Microbiology of the Czech Academy of Sciences, Vídeňská 1083, CZ 14220, Prague 4, Czech Republic

^2^ Department of Biochemistry and Microbiology, University of Chemistry and Technology Prague, Technická 3, CZ 16628 Prague 6, Czech Republic

**Contents**

Page

HPLC analysis of compounds **1**–**6** and their aglycones (**Table S1**) 3

Molecular docking of **2** to *An*Rut: hydrophobic interactions (**Table S2**) 4

Molecular docking of **2** to *An*Rut: hydrophobic interactions (**Table S3**) 5

Molecular docking of **1** to *An*Rut: π-π Interactions (**Table S4**) 6

Molecular docking of **1** to *An*Rut: π-π Interactions (**Table S5**) 7

Specific hydrolytic activities of *Mc*Glc and *Pc*Glc (**Table S6**) 8

^1^H and ^13^C NMR data of compound **6** (**Table S7**) 9

^1^H and ^13^C NMR data of compound **7** (**Table S8**) 10

^1^H and ^13^C NMR data of compound **8** (**Table S9**) 11

Superposition of *An*Rut, *Mc*Glc and *Pc*Glc structures (**Fig. S1**) 12

Hydrophobic and π-π interactions of bound compounds **1** and **2** (**Fig. S2**) 13

Glycoprotein staining of *Mc*Glc and *Pc*Glc (**Fig. S3**) 14

Activity-pH relationships (**Fig. S4**) 15

Activity-temperature relationships (**Fig. S5**) 16

Thermal stabilities (**Fig. S6**) 17

Initial velocity data of **5** (**Fig. S7**) 18

Initial velocity data: hydrolysis of **1** (**Fig. S8**) 19

Initial velocity data: hydrolysis of **2** (**Fig. S9**) 20

Initial velocity data: hydrolysis of **3** (**Fig. S10**) 21

Initial velocity data: hydrolysis of **4** (**Fig. S11**) 22

Enzymatic reactions with transglycosylation products: TLC analysis (**Fig. S12**) 23

Initial velocity data: hydrolysis of **2** in the presence of **6** (**Fig. S13**) 24

Transglycosylations with **2** in solution: varying the acceptor conc. (**Fig. S14**) 25

Time courses of transglycosylations in solution (*Mc*Glc, *Pc*Glc) (**Fig. S15**) 26

Time courses of transglycosylations in solution (*Mc*Glc) (**Fig. S16**) 27

Hydrolysis of **2** in suspension (**Fig. S17**) 28

Transglycosylations with 2-phenylethanol or 1,2-hexanediol (**Fig. S18**) 29

Mass spectrometry analysis of compound **6** (**Fig. S19**) 30

^1^H NMR spectrum of compound **6** (**Fig. S20**) 31

^13^C NMR spectrum of compound **6** (**Fig. S21**) 32

Mass spectrometry analysis of compounds **7**/**8** (**Fig. S22**) 33

^1^H NMR spectra of compounds **7**/**8** (**Fig. S23**) 34

^13^C NMR spectra of compounds **7**/**8** (**Fig. S24**) 35

Transglycosylations with **2** in suspension using *Mc*Glc (**Fig. S25**) 36

Transglycosylations with **2** in suspension using *Pc*Glc (**Fig. S26**) 37

Time courses of transglycosylations with **2** in suspension (**Fig. S27**) 38

Reaction scheme of retaining *Mc*Glc and *Pc*Glc (**Fig. S28**) 39

References 4

**Table S1** HPLC analysis of compounds **1**–**6** and their corresponding aglycones^a^

| Compound | λ [nm]^b^ | Retention time [min] |
| --- | --- | --- |
| **1** | 360 | 2.4 |
| **2** | 360 | 2.3 |
| Quercetin | 360 | 3.8 |
| **3** | 285 | 2.9 |
| Hesperetin | 285 | 4.8 |
| **4** | 370 | 2.8 |
| Isorhamnetin | 370 | 5.1 |
| **5** | 340 | 2.9 |
| Diosmetin | 340 | 4.9 |
| **6** | 257 | 2.0 |
| 2-Phenylethanol | 257 | 2.5 |

^a^ Gradients: 0–3 min, 7–30 % B; 3–5 min, 30 % B; 5–7 min, 30–7 % B; 7–7.5 min, 7 % B. Mobile phase A: CH_3_CN/H_2_O/HCO_2_H (5:95:0.1; v/v/v); mobile phase B: CH_3_CN/H_2_O/HCO_2_H (80:20:0.1; v/v/v)

^b^ Selected wavelength

**Table S2** Molecular docking of **2** to the active site of *An*Rut. Hydrophobic interactions between bound **2** and residues in the +1 subsite^a^

| *An*Rut atom | Atom in **2** | Distance [Å] | Interaction strength |
| --- | --- | --- | --- |
| 2035 CG LEU 162 | 5537 C6 | 4.90 | 0.676 |
| 2037 CD1 LEU 162 | 5535 C5 | 4.22 | 0.942 |
| 2037 CD1 LEU 162 | 5537 C6 | 3.93 | 0.780 |
| 2041 CD2 LEU 162 | 5537 C6 | 4.57 | 0.824 |
| 2055 CG2 THR 163 | 5590 C38 | 4.91 | 0.674 |
| 2906 CE MET 218 | 5532 C3 | 4.59 | 0.818 |
| 2906 CE MET 218 | 5534 C4 | 4.62 | 0.807 |
| 2906 CE MET 218 | 5535 C5 | 4.16 | 0.963 |
| 2906 CE MET 218 | 5537 C6 | 3.66 | 0.268 |
| 2951 CG PHE 221 | 5534 C4 | 4.86 | 0.672 |
| 2951 CG PHE 221 | 5535 C5 | 4.73 | 0.718 |
| 2954 CD2 PHE 221 | 5532 C3 | 4.61 | 0.758 |
| 2954 CD2 PHE 221 | 5534 C4 | 3.77 | 0.723 |
| 2954 CD2 PHE 221 | 5535 C5 | 3.70 | 0.594 |
| 2954 CD2 PHE 221 | 5537 C6 | 4.53 | 0.787 |
| 2954 CD2 PHE 221 | 5542 C10 | 4.67 | 0.737 |
| 2958 CE2 PHE 221 | 5534 C4 | 3.98 | 0.976 |
| 2958 CE2 PHE 221 | 5535 C5 | 3.57 | 0.346 |
| 2958 CE2 PHE 221 | 5537 C6 | 4.48 | 0.803 |
| 2958 CE2 PHE 221 | 5542 C10 | 4.71 | 0.723 |
| 2960 CZ PHE 221 | 5535 C5 | 4.54 | 0.784 |
| 2964 CA GLY 222 | 5542 C10 | 4.48 | 0.839 |
| 2964 CA GLY 222 | 5545 C13 | 4.01 | 0.901 |
| 2964 CA GLY 222 | 5548 C15 | 3.88 | 0.675 |
| 3574 CD1 PHE 261 | 5548 C15 | 4.66 | 0.741 |
| 3578 CE1 PHE 261 | 5542 C10 | 4.38 | 0.838 |
| 3578 CE1 PHE 261 | 5548 C15 | 4.71 | 0.725 |
| 3582 CZ PHE 261 | 5542 C10 | 4.84 | 0.680 |
| 3941 CB TYR 284 | 5542 C10 | 3.99 | 0.859 |
| 3941 CB TYR 284 | 5545 C13 | 3.95 | 0.800 |
| 3941 CB TYR 284 | 5548 C15 | 3.73 | 0.425 |
| 3944 CG TYR 284 | 5542 C10 | 4.09 | 0.940 |
| 3944 CG TYR 284 | 5545 C13 | 4.84 | 0.681 |
| 3944 CG TYR 284 | 5548 C15 | 4.41 | 0.828 |
| 3947 CD2 TYR 284 | 5542 C10 | 3.63 | 0.468 |
| 3947 CD2 TYR 284 | 5548 C15 | 4.11 | 0.932 |
| 3951 CE2 TYR 284 | 5542 C10 | 4.39 | 0.835 |
| 3991 CE1 TYR 286 | 5532 C3 | 3.95 | 0.985 |
| 3991 CE1 TYR 286 | 5534 C4 | 4.79 | 0.698 |
| 3993 CE2 TYR 286 | 5532 C3 | 4.65 | 0.743 |
| 3993 CE2 TYR 286 | 5534 C4 | 4.86 | 0.672 |
| 4007 CG GLU 287 | 5532 C3 | 4.86 | 0.693 |
| 4007 CG GLU 287 | 5545 C13 | 4.93 | 0.664 |
| 5338 CH2 TRP 374 | 5563 C24 | 4.81 | 0.711 |

^a^ See Fig. S3 for the numbering of the substrate atoms

**Table S3** Molecular docking of **2** to the active site of *An*Rut. π-π Interactions between bound **2** and residues in the +1 subsite^a^

| *An*Rut atom | Atom in **2** | Distance [Å] | Interaction strength |
| --- | --- | --- | --- |
| 2954 CD2 PHE 221 | 5535 C5 | 3.70 | 0.844 |
| 2954 CD2 PHE 221 | 5537 C6 | 4.53 | 0.532 |
| 2954 CD2 PHE 221 | 5540 O8 | 4.01 | 1.000 |
| 2958 CE2 PHE 221 | 5537 C6 | 4.48 | 0.181 |
| 2958 CE2 PHE 221 | 5540 O8 | 4.50 | 1.000 |
| 2960 CZ PHE 221 | 5544 C12 | 4.90 | 0.524 |
| 3574 CD1 PHE 261 | 5550 C16 | 4.31 | 0.902 |
| 3578 CE1 PHE 261 | 5543 C11 | 4.48 | 1.000 |
| 3578 CE1 PHE 261 | 5548 C15 | 4.71 | 0.105 |
| 3578 CE1 PHE 261 | 5550 C16 | 3.97 | 1.000 |
| 3582 CZ PHE 261 | 5543 C11 | 4.75 | 0.125 |
| 3947 CD2 TYR 284 | 5543 C11 | 3.70 | 0.111 |
| 3947 CD2 TYR 284 | 5548 C15 | 4.11 | 0.140 |
| 3947 CD2 TYR 284 | 5550 C16 | 3.45 | 1.000 |
| 3951 CE2 TYR 284 | 5543 C11 | 3.97 | 1.000 |
| 3951 CE2 TYR 284 | 5544 C12 | 4.89 | 0.253 |
| 3951 CE2 TYR 284 | 5550 C16 | 4.37 | 1.000 |
| 3991 CE1 TYR 286 | 5531 C2 | 4.38 | 1.000 |
| 3991 CE1 TYR 286 | 5532 C3 | 3.95 | 0.344 |
| 3993 CE2 TYR 286 | 5532 C3 | 4.65 | 0.485 |
| 3993 CE2 TYR 286 | 5540 O8 | 4.22 | 1.000 |
| 3995 CZ TYR 286 | 5531 C2 | 4.41 | 1.000 |

^a^ See Fig. S3 for the numbering of the substrate atoms

**Table S4** Molecular docking of **1** to the active site of *An*Rut. Hydrophobic interactions between bound **1** and residues in the +1 subsite^a^

| *An*Rut atom | Atom in **2** | Distance [Å] | Interaction strength |
| --- | --- | --- | --- |
| 2035 CG LEU 162 | 5537 C6 | 4.69 | 0.759 |
| 2037 CD1 LEU 162 | 5535 C5 | 4.04 | 0.982 |
| 2037 CD1 LEU 162 | 5537 C6 | 3.76 | 0.455 |
| 2041 CD2 LEU 162 | 5537 C6 | 4.33 | 0.904 |
| 2906 CE MET 218 | 5532 C3 | 4.53 | 0.836 |
| 2906 CE MET 218 | 5534 C4 | 4.62 | 0.836 |
| 2906 CE MET 218 | 5535 C5 | 4.06 | 0.997 |
| 2906 CE MET 218 | 5537 C6 | 3.55 | 0.063 |
| 2951 CG PHE 221 | 5534 C4 | 4.90 | 0.657 |
| 2951 CG PHE 221 | 5535 C5 | 4.74 | 0.715 |
| 2954 CD2 PHE 221 | 5532 C3 | 4.70 | 0.728 |
| 2954 CD2 PHE 221 | 5534 C4 | 3.83 | 0.839 |
| 2954 CD2 PHE 221 | 5535 C5 | 3.74 | 0.679 |
| 2954 CD2 PHE 221 | 5537 C6 | 4.57 | 0.773 |
| 2954 CD2 PHE 221 | 5542 C10 | 4.51 | 0.793 |
| 2954 CD2 PHE 221 | 5545 C13 | 4.88 | 0.666 |
| 2958 CE2 PHE 221 | 5534 C4 | 4.00 | 0.970 |
| 2958 CE2 PHE 221 | 5535 C5 | 3.59 | 0.385 |
| 2958 CE2 PHE 221 | 5537 C6 | 4.51 | 0.793 |
| 2958 CE2 PHE 221 | 5542 C10 | 4.56 | 0.777 |
| 2960 CZ PHE 221 | 5535 C5 | 4.50 | 0.796 |
| 2964 CA GLY 222 | 5542 C10 | 4.50 | 0.833 |
| 2964 CA GLY 222 | 5545 C13 | 3.79 | 0.531 |
| 2964 CA GLY 222 | 5548 C15 | 3.72 | 0.406 |
| 3574 CD1 PHE 261 | 5548 C15 | 4.65 | 0.744 |
| 3578 CE1 PHE 261 | 5542 C10 | 4.42 | 0.826 |
| 3578 CE1 PHE 261 | 5548 C15 | 4.66 | 0.742 |
| 3582 CZ PHE 261 | 5542 C10 | 4.82 | 0.687 |
| 3941 CB TYR 284 | 5542 C10 | 4.10 | 0.987 |
| 3941 CB TYR 284 | 5545 C13 | 4.35 | 0.891 |
| 3941 CB TYR 284 | 5548 C15 | 3.79 | 0.524 |
| 3944 CG TYR 284 | 5542 C10 | 4.19 | 0.903 |
| 3944 CG TYR 284 | 5548 C15 | 4.47 | 0.806 |
| 3947 CD2 TYR 284 | 5542 C10 | 3.76 | 0.711 |
| 3947 CD2 TYR 284 | 5548 C15 | 4.19 | 0.903 |
| 3951 CE2 TYR 284 | 5542 C10 | 4.48 | 0.803 |
| 3991 CE1 TYR 286 | 5532 C3 | 3.99 | 0.972 |
| 3991 CE1 TYR 286 | 5534 C4 | 4.85 | 0.676 |
| 3993 CE2 TYR 286 | 5532 C3 | 4.75 | 0.712 |
| 3993 CE2 TYR 286 | 5534 C4 | 4.98 | 0.631 |
| 4007 CG GLU 287 | 5545 C13 | 4.85 | 0.697 |
| 5338 CH2 TRP 374 | 5563 C24 | 4.95 | 0.659 |

^a^ See Fig. S3 for the numbering of the substrate atoms

**Table S5** Molecular docking of **1** to the active site of *An*Rut. π-π Interactions between bound **1** and residues in the +1 subsite^a^

| *An*Rut atom | Atom in **2** | Distance [Å] | Interaction strength |
| --- | --- | --- | --- |
| 2954 CD2 PHE 221 | 5535 C5 | 3.77 | 0.816 |
| 2954 CD2 PHE 221 | 5537 C6 | 4.57 | 0.490 |
| 2954 CD2 PHE 221 | 5540 O8 | 3.86 | 1.000 |
| 2958 CE2 PHE 221 | 5537 C6 | 4.51 | 0.195 |
| 2958 CE2 PHE 221 | 5540 O8 | 4.35 | 1.000 |
| 2960 CZ PHE 221 | 5544 C12 | 4.80 | 0.578 |
| 3574 CD1 PHE 261 | 5550 C16 | 4.40 | 0.811 |
| 3578 CE1 PHE 261 | 5543 C11 | 4.53 | 1.000 |
| 3578 CE1 PHE 261 | 5548 C15 | 4.66 | 0.077 |
| 3578 CE1 PHE 261 | 5550 C16 | 4.02 | 1.000 |
| 3582 CZ PHE 261 | 5543 C11 | 4.76 | 0.120 |
| 3947 CD2 TYR 284 | 5543 C11 | 3.82 | 0.155 |
| 3947 CD2 TYR 284 | 5550 C16 | 3.46 | 1.000 |
| 3951 CE2 TYR 284 | 5543 C11 | 4.07 | 1.000 |
| 3951 CE2 TYR 284 | 5550 C16 | 4.37 | 1.000 |
| 3991 CE1 TYR 286 | 5531 C2 | 4.54 | 0.994 |
| 3991 CE1 TYR 286 | 5532 C3 | 3.99 | 0.103 |
| 3993 CE2 TYR 286 | 5532 C3 | 4.75 | 0.534 |
| 3993 CE2 TYR 286 | 5540 O8 | 4.30 | 1.000 |
| 3995 CZ TYR 286 | 5531 C2 | 4.56 | 1.000 |

^a^ See Fig. S3 for the numbering of the substrate atoms

**Table S6** Specific hydrolytic activities of *Mc*Glc and *Pc*Glc for compounds **1**, **2**, **9** and **10**. The activities for **2** were determined in the presence or absence of glucose, rhamnose or rutinose

| Compound^a^ | Added carbohydrate^a^ | Specific activity^b^  [μmol min^–1^ mg^–1^] | |
| --- | --- | --- | --- |
|  |  | *Mc*Glc | *Pc*Glc |
| **1** | – | 69.8 ± 5 | 42.8 ± 7 |
| **2** | – | 31 ± 2 | 55 ± 4 |
|  | β-D-glucose | 30 ± 3 | 52 ± 3 |
|  | α-L-rhamnose | 30 ± 3 | 52 ± 4 |
|  | rutinose | 30 ± 3 | 53 ± 5 |
| **9** | – | 0.2 ± 0.1 | 0.2 ± 0.1 |
| **10** |  | 0.1 ± 0.05 | 1.1 ± 0.5 |

^a^ Concentration: 2.0 mM

^b^ The measurements were performed in triplicate

**Table S7** ^1^H and ^13^C NMR data of compound **6** (399.87 MHz for ^1^H, 100.55 MHz for ^13^C, D_2_O, 30 ^o^C)

|  | **Atom** | ***δ*_C_** | **m.** | ***δ*_H_** | **n_H_** | **m.** | ***J* [Hz]** |
| --- | --- | --- | --- | --- | --- | --- | --- |
| **Glc** | **1** | 102.62 | D | 4.461 | 1 | d | 8.0 |
|  | **2** | 73.29 | D | 3.253 | 1 | dd | 9.3, 8.0 |
|  | **3** | 75.97 | D | 3.471 | 1 | dd | 9.3, 9.1 |
|  | **4** | 69.97 | D | 3.391 | 1 | dd | 9.7, 9.1 |
|  | **5** | 75.00 | D | 3.553 | 1 | ddd | 9.7, 6.2, 2.0 |
|  | **6** | 67.15 | T | 3.999 | 1 | dd | 11.6, 2.0 |
|  |  |  |  | 3.695 | 1 | dd | 11.6, 6.2 |
| **Rha** | **1‘** | 100.87 | D | 4.834 | 1 | dm | 1.8 |
|  | **2‘** | 70.33 | D | 3.977 | 1 | dd | 3.5, 1.8 |
|  | **3‘** | 70.47 | D | 3.792 | 1 | dd | 9.8, 3.5 |
|  | **4‘** | 72.31 | D | 3.444 | 1 | dd | 9.8, 9.5 |
|  | **5‘** | 68.94 | D | 3.754 | 1 | dq | 9.8, 6.3 |
|  | **6‘** | 16.93 | Q | 1.295 | 3 | d | 6.3 |
| **aglycone** | **1“** | 71.17 | T | 4.130 | 1 | ddd | 10.2, ΣJ = 13.8 |
|  |  |  |  | 3.941 | 1 | ddd | 10.2, ΣJ = 14.0 |
|  | **2“** | 35.54 | T | 2.981 | 2 | dd | ΣJ = 13.9 |
|  | ***ipso-*** | 138.98 | S | - | 0 | - |  |
|  | ***ortho-*** | 129.27 | D | 7.375  . | 2 | m |  |
|  | ***meta-*** | 128.95 | D | 7.400 | 2 | m |  |
|  | ***para-*** | 126.84 | D | 7.312 | 1 | m |  |

**Table S8** ^1^H and ^13^C NMR data of the major diastereomer of compound **7** (700.13 MHz for ^1^H, 176.05 MHz for ^13^C, D_2_O and MeOD (9:1, v/v), 30 ^o^C)

| **A** | **Atom** | **δ_C_** | **m.** | **δ_H_** | **n_H_** | **m.** | **J[Hz]** | **HMBC^x^** |
| --- | --- | --- | --- | --- | --- | --- | --- | --- |
| **Glc** | **1** | 103.21 | D | 4.377 | 1 | d | 7.9 | 1” |
|  | **2** | 73.47 | D | 3.251 | 1 | dd | 9.6, 7.9 |  |
|  | **3** | 75.90 | D | 3.425 | 1 | dd | 9.6, 8.9 |  |
|  | **4** | 69.95 | D | 3.352 | 1 | dd | 9.9, 8.9 |  |
|  | **5** | 74.96 | D | 3.499 | 1 | ddd | 9.9, 5.8, 2.0 |  |
|  | **6** | 66.95 | T | 3.941 | 1 | dd | 11.6, 2.0 | 1’ |
|  |  |  |  | 3.646 | 1 | dd | 11.6, 5.8 |  |
| **Rha** | **1’** | 100.81 | D | 4.758 | 1 | d | 1.8 | 6 |
|  | **2’** | 70.35 | D | 3.905 | 1 | dd | 3.5, 1.8 |  |
|  | **3’** | 70.51 | D | 3.717 | 1 | dd | 9.6, 3.5 |  |
|  | **4’** | 72.33 | D | 3.379 | 1 | dd | 9.6, 9.6 |  |
|  | **5’** | 68.85 | D | 3.679 | 1 | dq | 9.6, 6.3 |  |
|  | **6’** | 16.90 | Q | 1.246 | 3 | d | 6.3 |  |
| **aglycone** | **1”** | 74.55 | T | 3.856 | 1 | dd | 10.7, 3.1 | 1 |
|  |  |  |  | 3.487 | 1 | dd | 10.7, 7.8 |  |
|  | **2”** | 70.75 | D | 3.795 | 1 | m | Σ*J* = 23.8 |  |
|  | **3”** | 32.27 | T | 1.46^H^ | 1 | m |  |  |
|  |  |  |  | 1.41^H^ | 1 | m |  |  |
|  | **4”** | 27.16 | T | 1.34^H^ | 1 | m |  |  |
|  |  |  |  | 1.27^H^ | 1 | m |  |  |
|  | **5”** | 22.22 | T | 1.28^H^ | 2 | m |  |  |
|  | **6”** | 13.41 | Q | 0.847 | 3 | t | 7.1 |  |

^H^ … HSQC readout; ^x^ … diagnostic C to H correlations

The approximate molar ratio of both pairs of epimers (diastereomers) in the **7**/**8** mixture is

7^major^ : 8^major^ : 7^minor^ : 8^minor^ = 56 : 21 : 14 : 9.

**Table S9** ^1^H and ^13^C NMR data of the major diastereomer of compound **8** (700.13 MHz for ^1^H, 176.05 MHz for ^13^C, D_2_O and MeOD (9:1, v/v), 30 ^o^C)

| **B** | **Atom** | **δ_C_** | **m.** | **δ_H_** | **n_H_** | **m.** | **J[Hz]** | **HMBC^x^** |
| --- | --- | --- | --- | --- | --- | --- | --- | --- |
| **Glc** | **1** | 102.53 | D | 4.473 | 1 | d | 7.9 | 2” |
|  | **2** | 73.66 | D | 3.227 | 1 | dd | 9.6, 7.9 |  |
|  | **3** | 76.09 | D | 3.420 | 1 | dd | 9.6, 8.9 |  |
|  | **4** | 69.92 | D | 3.358 | 1 | dd | 9.8, 8.9 |  |
|  | **5** | 74.89 | D | 3.473 | 1 | ddd | 9.8, 6.0, 1.9 |  |
|  | **6** | 66.87 | T | 3.934 | 1 | dd | 11.4, 1.9 | 1’ |
|  |  |  |  | 3.598 | 1 | dd | 11.4, 6.0 |  |
| **Rha** | **1’** | 100.74 | D | 4.747 | 1 | dm | 1.8 | 6 |
|  | **2’** | 70.38 | D | 3.896 | 1 | dd | 3.5, 1.8 |  |
|  | **3’** | 70.53^a^ | D | 3.725 | 1 | dd | 9.6, 3.5 |  |
|  | **4’** | 72.37^a^ | D | 3.38^H^ | 1 | m |  |  |
|  | **5’** | 68.83 | D | 3.66^H^ | 1 | m |  |  |
|  | **6’** | 16.92 | Q | 1.249 | 3 | d | 6.3 |  |
| **aglycone** | **1”** | 63.59 | T | 3.647 | 1 | dd | 12.3, 3.7 |  |
|  |  |  |  | 3.544 | 1 | dd | 12.3, 5.8 |  |
|  | **2”** | 81.75 | D | 3.753 | 1 | m |  | 1 |
|  | **3”** | 30.79 | T | 1.54^H^ | 2 | m |  |  |
|  | **4”** | 27.00 | T | 1.31^H^  . | 2 | m |  |  |
|  | **5”** | 22.11^a^ | T | 1.29^H^ | 2 | m |  |  |
|  | **6”** | 13.45^a^ | Q | 0.852 | t | 7.1 |  |  |

^H^ … HSQC readout; ^a^ … tentative assignment; ^x^ … diagnostic C to H correlations


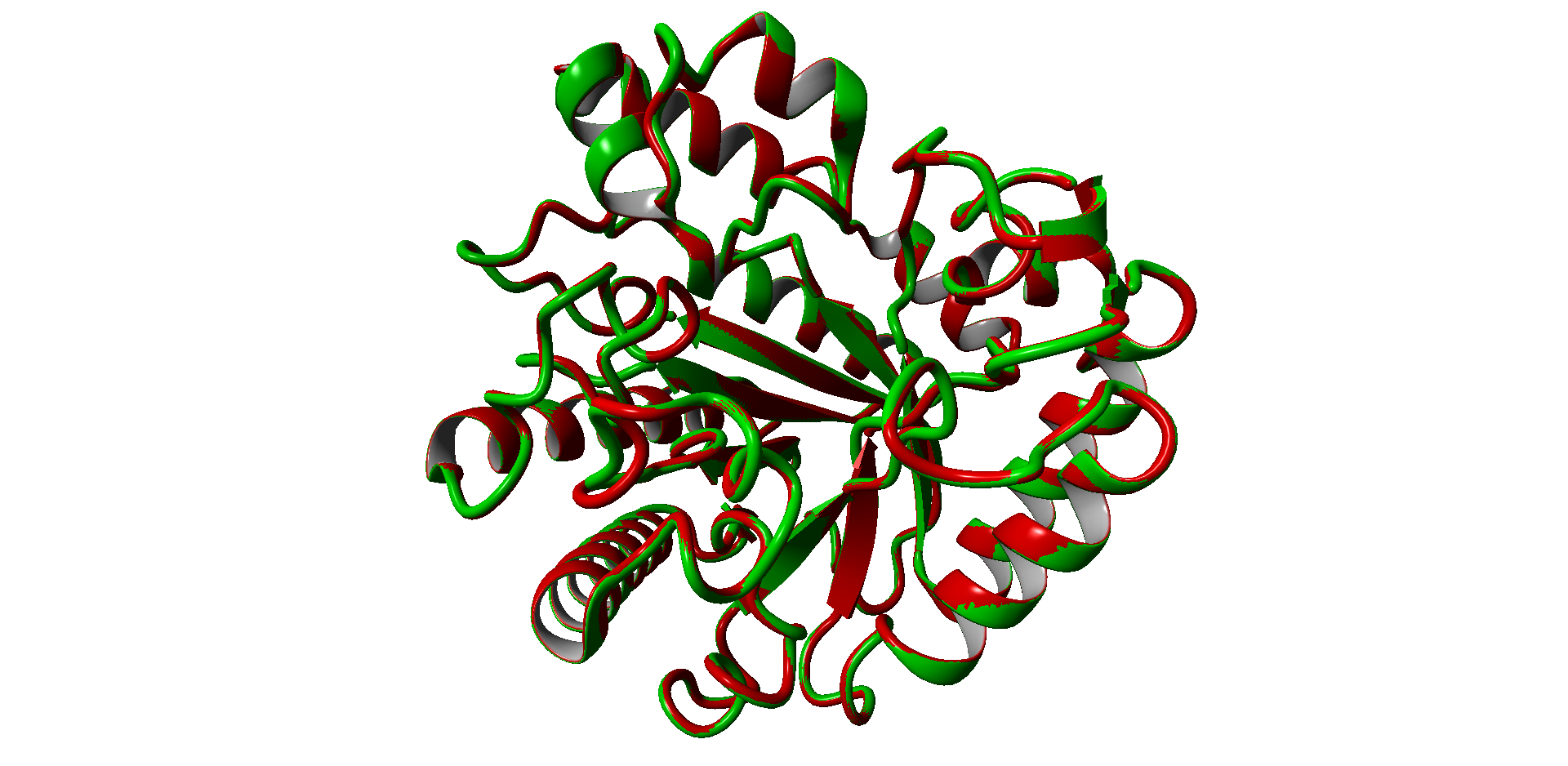


**a**


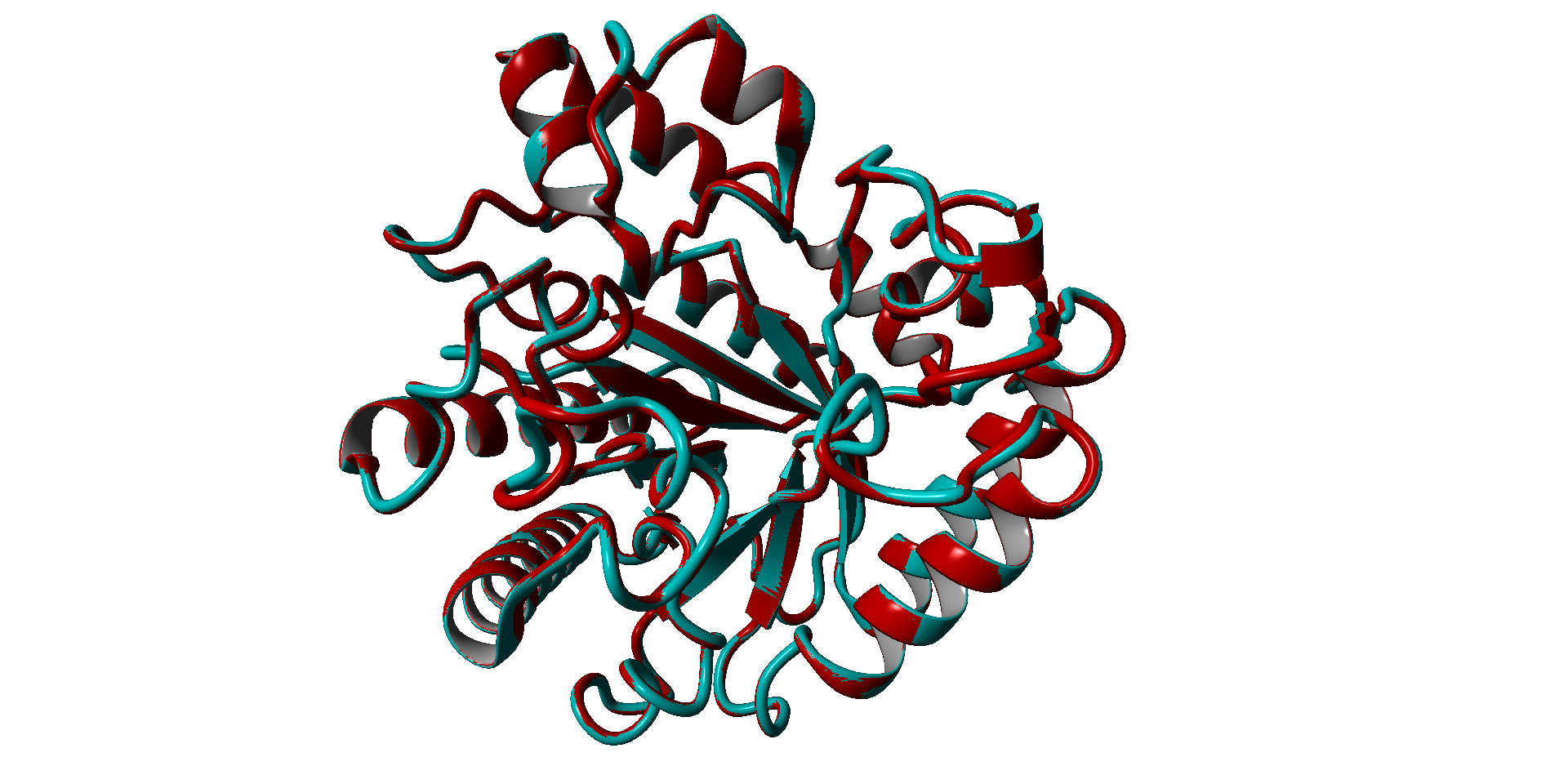


**b**

**c**

*An*Rut (210, 319) **PQSYT**LE**PINEP**A**DN**NTNMVV **G**T**GKFP**V**FVGEWAI**QATYN**N**T

*Mc*Glc (231, 339) **PQSYT**IE**PINEP**V**DN**-HDFST **G**D**GKFP**T**FVGEWAI**QTGGK**N**E

*Pc*Glc (219, 327) **PQSYT**LS**PINEP**V**DN**-RDFST **G**D**GKFP**T**FVGEWAI**EAS-D**N**D

**Fig. S1** Superposition of the ribbon diagrams of the experimentally determined *An*Rut structure in red (Pachl et al. 2020) and the modelled structures of *Mc*Glc and *Pc*Glc. The coordinates of the *An*Rut X-ray structure were used as a template (NCBI accession number 6I1A). The model quality estimates GMQE and QMEAN Z-score are given for each model (Benkert et al. 2011). **a** Modelled structure of *Mc*Glc in green; GMQE: 0.78; QMEAN Z-score: –0.74. **b** Modelled structure of *Pc*Rut in blue; GMQE: 0.80; QMEAN Z-score: –0.35. **c** Sequence alignment of *An*Rut with *Mc*Glc and *Pc*Glc. Only the two segments around the acid/base catalyst (in red: E210, E231 and E219 for *An*Rut, *Mc*Glc and *Pc*Glc, respectively) and the catalytic nucleophile (in orange: E319, E339 and E327 for *An*Rut, *Mc*Glc and *Pc*Glc, respectively) are shown. The numbering refers to the translated nucleotide sequences

**
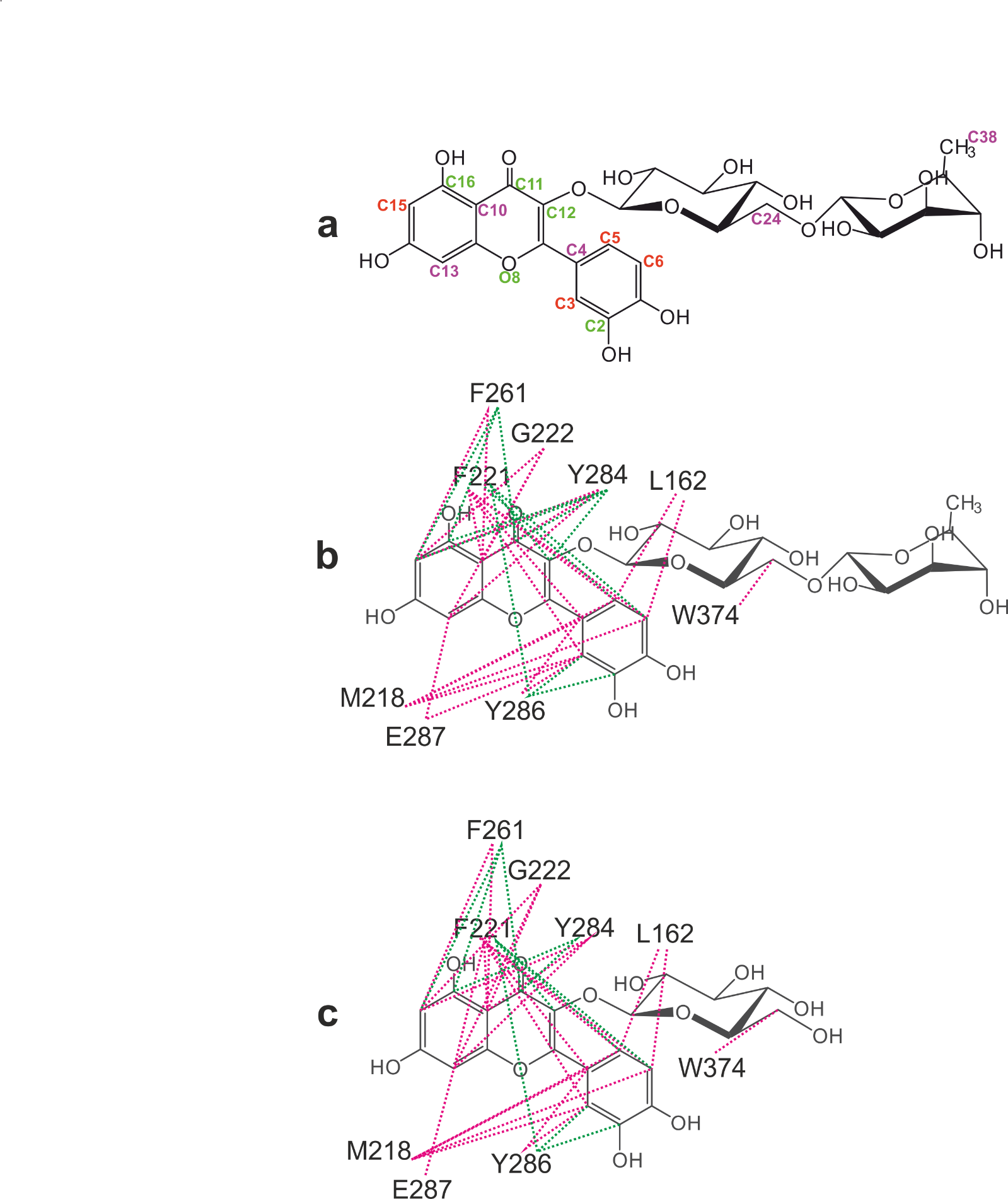
**

**Fig. S2** Visualization of hydrophobic and π-π interactions between the side chains of *An*Rut and bound substrates **1** or **2** based on the molecular docking analysis (Tables S2–S5). **a** Atom labelling in **2**; substrate atoms that were involved in hydrophobic interactions are labelled in magenta; atoms that were involved in π-π interactions are labelled in green; atoms that were involved in both types of interactions are indicated in red. **b** Hydrophobic (dashed lines in magenta) and π-π interactions (green lines) between *An*Rut and compound **2**. **c** Hydrophobic and π-π interactions between *An*Rut and compound **1**

**
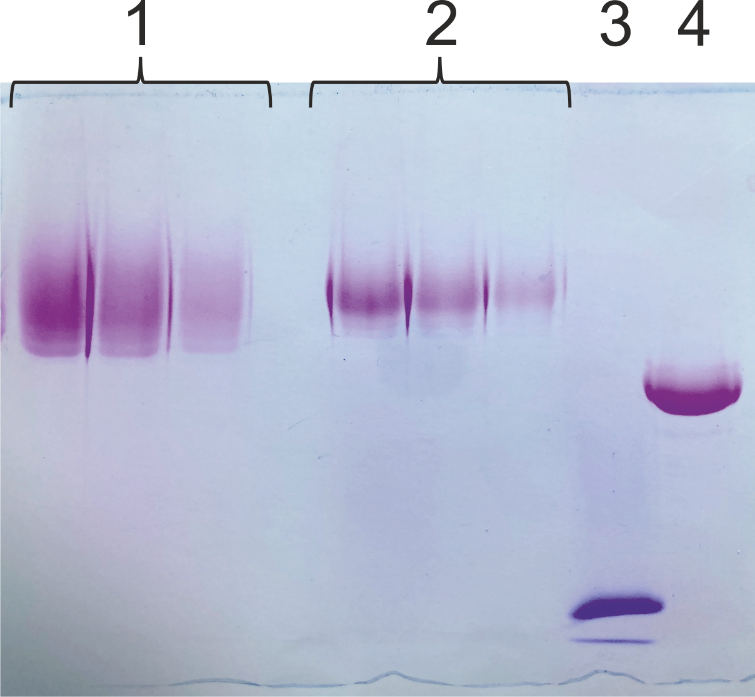
**

**Fig. S3** SDS-PAGE analysis of purified, heterologously produced *Mc*Glc (with a polypeptide mass of 46.1 kDa) and *Pc*Glc (with a polypeptide mass of 42.5 kDa) for the presence of carbohydrate moieties attached to the polypeptide chains. Glycoproteins are colored magenta. (1) *Pc*Glc and (2) *Mc*Glc with 5, 10 and 15 µg of protein loaded per lane. (3) Soybean trypsin inhibitor as a negative control in blue (~20 kDa); visualized only in the Coomassie staining step, which followed the staining of the glycoproteins. (4) Horseradish peroxidase as a positive control (~44 kDa, which includes the polypeptide chain of 33.9 kDa)

**Fig. S4** Activity-pH relationships of *Mc*Glc and *Pc*Glc for hydrolysis of **2**. The 15-min reactions were performed at 48 °C by adding **2** to a final concentration of 2.7 mM in the presence of 10 % dimethyl sulfoxide after a 30-min incubation of *Mc*Glc (○) or *Pc*Glc (□) at 35 °C. Citrate buffers (50 mM) were used. The measurements were performed in triplicate

**Fig. S5** Activity-temperature relationships for hydrolysis of **2** using *Mc*Glc or *Pc*Glc. The reaction conditions were as follows: 10 % dimethyl sulfoxide, 2.7 mM of compound **2**, pH 3.5 for *Mc*Glc (○), or pH 5.0 for *Pc*Glc (□). The reactions were stopped after 15 min of incubation at a given temperature. The measurements were performed in triplicate

**Fig. S6** Thermal stabilities of *Mc*Glc and *Pc*Glc. The enzyme samples containing *Mc*Glc (○) or *Pc*Glc (□) were incubated at different temperatures (20–75 °C) for 10 min at pH 4.0 or 5.0, respectively. The residual activity was determined at 30 °C in the presence of 10 % (v/v) dimethyl sulfoxide after adding compound **2** (final concentration of 5.0 mM)

**a**

**b**

**Fig. S7** Initial velocity data with varying concentrations of compound **5**. Reaction conditions: 48 °C, 50 mM citrate buffer. **a** *Mc*Glc (pH 4.0); **b** *Pc*Glc (pH 5.0)

**a**

**b**

**Fig. S8** Initial velocity data with varying concentrations of compound **1**. Reaction conditions: 48 °C, 50 mM citrate buffer. **a** *Mc*Glc (pH 4.0); **b** *Pc*Glc (pH 5.0)

**a**

**b**

**Fig. S9** Initial velocity data with varying concentrations of compound **2**. Reaction conditions: 48 °C, 50 mM citrate buffer. **a** *Mc*Glc (pH 4.0); **b** *Pc*Glc (pH 5.0)

**a**

**b**

**Fig. S10** Initial velocity data with varying concentrations of compound **3**. Reaction conditions: 48 °C, 50 mM citrate buffer. **a** *Mc*Glc (pH 4.0); **b** *Pc*Glc (pH 5.0)

**a**

**b**

**Fig. S11** Initial velocity data with varying concentrations of compound **4**. Reaction conditions: 48 °C, 50 mM citrate buffer. **a** *Mc*Rut (pH 4.0); **b** *Pc*Glc (pH 5.0)


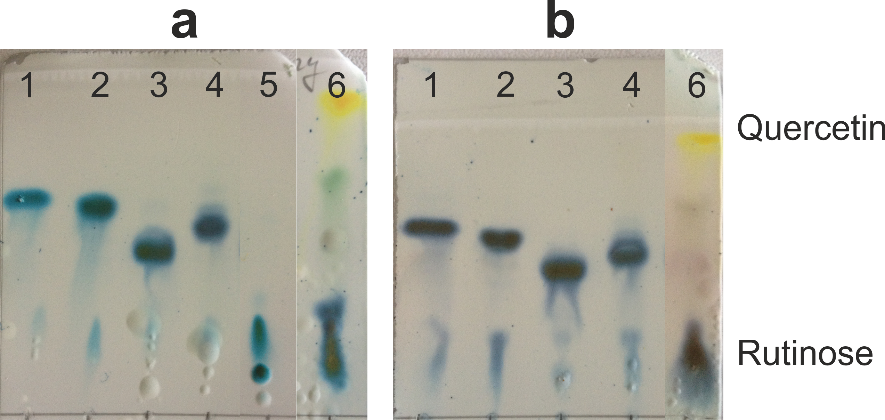


**Fig. S12** Thin-layer chromatography: analysis of enzymatic reactions containing selected transglycosylation products as substrates. *Mc*Glc as a catalyst (**a**); *Pc*Glc as a catalyst (**b**). Highly limited hydrolysis of pentyl rutinoside (lane 1), compound **6** (lane 2), 2-azidoethyl rutinoside (lane 3), and compound **7**/**8** (lane 4). Plain rutinose as a control (lane 5). The hydrolysis products were visualized using the anthrone reagent 24 h after the initiation of the reaction. Control reaction with compound **2** as a substrate, analyzed after 2 h of reaction (lane 6). The formation of quercetin and rutinose is indicated. Rutinose and the unreacted transglycosylation products are colored blue. Quercetin and residual **2** in the control reactions are colored yellow and green, respectively. Reaction conditions: 37 °C, McIlvaine buffer (pH 4.0 for *Mc*Glc-based reactions or pH 5.0 for *Pc*Glc-based reactions), 15 mM transglycosylation product, 0.12 µg mL^–1^ enzyme. Pentyl rutinoside and 2-azidoethyl rutinoside were generated using *An*Rut (Brodsky et al. 2020)

**a**

**b**

**Fig. S13** Initial velocity data of hydrolysis reactions with **2** as a substrate in the presence of various concentrations of compound **6**. Three different concentrations of **2** were used: 50 µM (□), 200 µM (○) and 750 µM (◇). The initial velocity measurements were performed in triplicate with either *Mc*Glc (**a**) or *Pc*Glc (**b**) as catalysts. **a** 48 °C, McIlvaine buffer (pH 4.0), 10 % (v/v) dimethyl sulfoxide. **b** 48 °C, McIlvaine buffer (pH 5.0), 10 % (v/v) dimethyl sulfoxide

**Fig. S14** Transglycosylation reactions in the presence of various acceptor concentrations (2-phenylethanol). The formation of quercetin (○, ●) and compound **6** (□, ■) was determined after 3 h of reaction at 35 °C in the presence of 100 mM **2**, 25 % dimethyl sulfoxide, and *Mc*Glc (open symbols) or *Pc*Glc (filled symbols). The S/E ratio was 67'000 mol mol^–1^ for both enzymes. The reactions were performed in McIlvaine buffer at pH 4.0 or 5.0 for the *Mc*Glc or *Pc*Glc-based reactions, respectively. The measurements were performed in duplicate

**a**

**b**

**Fig. S15** Time courses of transglycosylation reactions with 2-phenylethanol as an acceptor and **2** as a glycosyl donor. The concentrations of quercetin (○) and transglycosylation product **6** (□) are shown. The reactions were performed in triplicate in McIlvaine buffer (pH 4.0 or 5.0) at 35 °C in the presence of 25 % (v/v) dimethyl sulfoxide and 100 mM **2** using an S/E ratio of 67'000 mol mol^–1^ with 200 mM 2-phenylethanol and *Mc*Glc (**a**) or 400 mM 2-phenylethanol and *Pc*Glc (**b**)

**a**

**b**

**Fig. S16** Time courses of transglycosylation reactions with *Mc*Glc as a catalyst, 2-phenylethanol as an acceptor and **2** as a glycosyl donor. The concentrations of quercetin (○) and transglycosylation product **6** (□) are shown. The reactions were performed in triplicate in McIlvaine buffer (pH 4.0) at 35 °C in the presence of 25 % (v/v) dimethyl sulfoxide, 100 mM **2**, and 400 mM 2-phenylethanol, using an S/E ratio of 67'000 (**a**) or 20'000 mol mol^–1^ (**b**)

**Fig. S17** Quercetin production using *Mc*Glc or *Pc*Glc in suspensions containing **2**. Initial concentration of **2**: 185 g L^-1^ (300 mM). Purified *Mc*Glc (○); purified *Pc*Glc (□). Reaction conditions: 35 °C, 0.34 mg mL^-1^ of enzyme, McIlvaine buffer at pH 4.0 (*Mc*Glc) or pH 5.0 (*Pc*Glc)


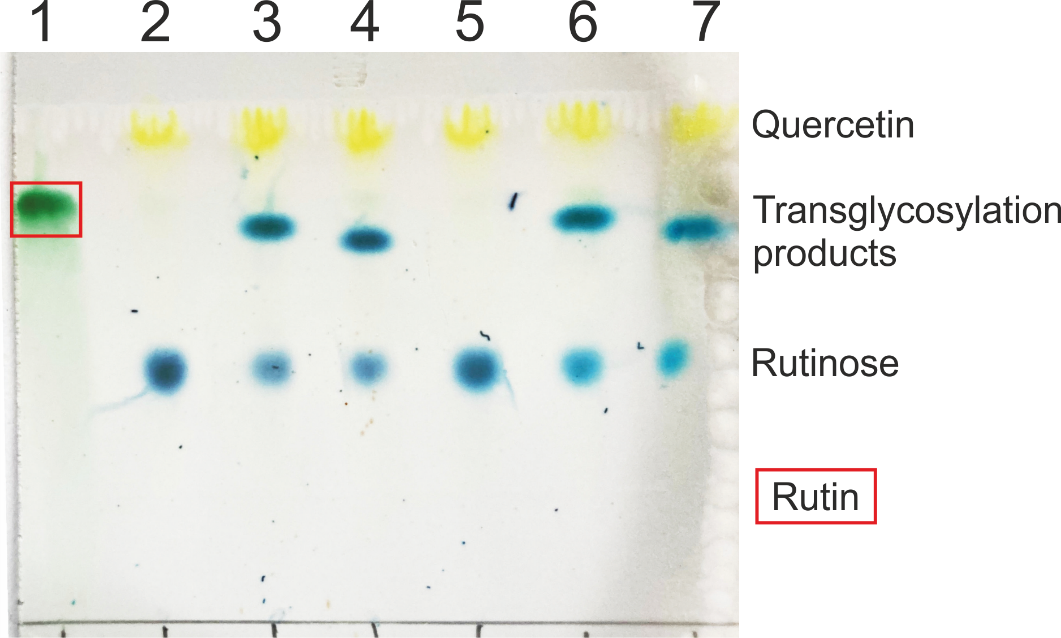


**Fig. S18** TLC analysis of transglycosylation reactions using *Mc*Glc or *Pc*Glc as a catalyst, compound **2** as a rutinosyl donor and 2-phenylethanol or 1,2-hexanediol as an acceptor. The 20-h reactions were performed at 35 °C in 50-mM citrate buffer (pH 4.0 for *Mc*Glc or pH 5.0 for *Pc*Glc) with **2** at a concentration of 200 mM. The transglycosylation reactions were performed with either 2-phenylethanol (516 mM; lanes 3 and 6) or 1,2-hexanediol (496 mM; lanes 4 and 7) in the presence of 1.67 mg ml^–1^ of *Mc*Glc or *Pc*Glc. Lane 1: no addition of enzyme; lanes 2–4: *Mc*Glc as a catalyst; lanes 5–7: *Pc*Glc as a catalyst. Lanes 2 and 5: no acceptor added. The reaction products were visualized using the anthrone reagent. Mobile phase: ethyl acetate/2-propanol/water (3:2:2; v/v/v). The quercetin spots are colored yellow; spots of rutinose and compounds **6**, **7** and **8** are colored blue. Residual **2** is colored green and boxed


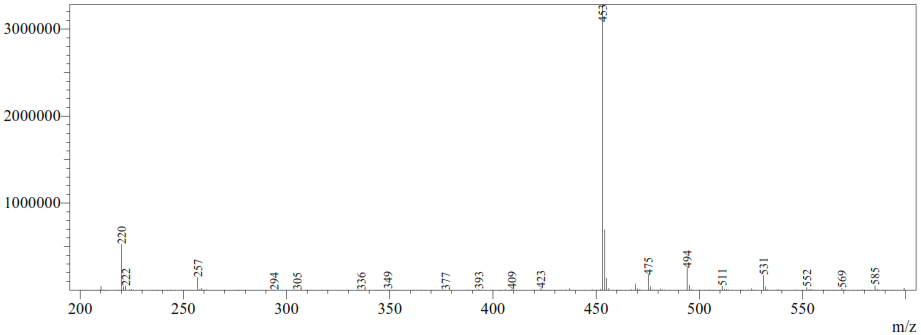


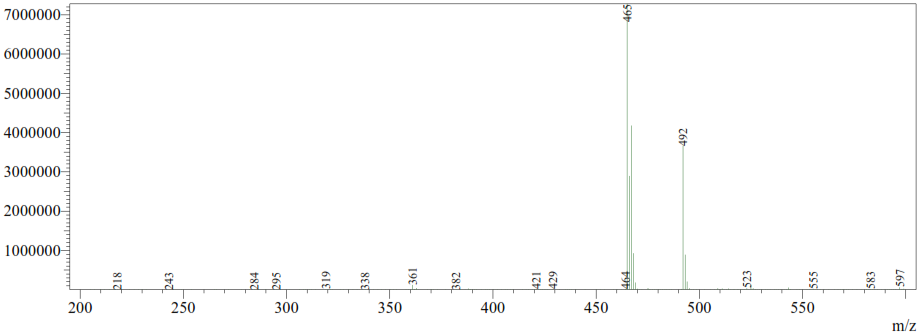


**Fig. S19** Mass spectrometry (MS) analysis of compound **6** generated in a transglycosylation reaction with **2** as a glycosyl donor, 2-phenylethanol as an acceptor and *Mc*Glc as a catalyst. The preparative transglycosylation reaction was performed as described in the Materials and methods section. Upper MS spectrum (ESI^+^): *m*/*z* = 453 [M+Na]^+^, calculated mass for C_20_H_30_O_10_Na: 453.4. Lower MS spectrum (ESI^–^): *m*/*z* = 465 [M+Cl]^–^, calculated mass for C_20_H_29_O_10_Cl: 464.9; *m*/*z* = 492 [M+NO_3_]^–^, calculated mass for C_20_H_29_O_13_N: 491.4. Mass spectra were recorded with a Shimadzu Prominence LC analytical system (Shimadzu, Japan) consisting of a Shimadzu CBM-20A system controller, Shimadzu LC-20AD binary HPLC pump, a Shimadzu CTO-10AS column oven, a Shimadzu SIL-20ACHT cooling autosampler and a Shimadzu SPD-20MA diode array detector. The samples were dissolved in acetonitrile. The mobile phase was composed of acetonitrile; the flow rate was 0.3 mL min^–1^ (25 °C); the injection volume was 1 μL. The MS-ESI parameters were as follows: positive and negative mode; ESI interface voltage: 4.5 kV, –3.5 kV; detector voltage: 1.15 kV; nebulizing gas flow: 1.5 mL min^–1^; drying gas flow: 15 mL min^–1^; heat block temperature: 200 °C; temperature of desolvation line pipe: 250 °C; SCAN mode: 300–600 *m*/*z*. The spectra were analyzed using the software LabSolutions (ver. 5.75 SP2; Shimadzu, Japan)


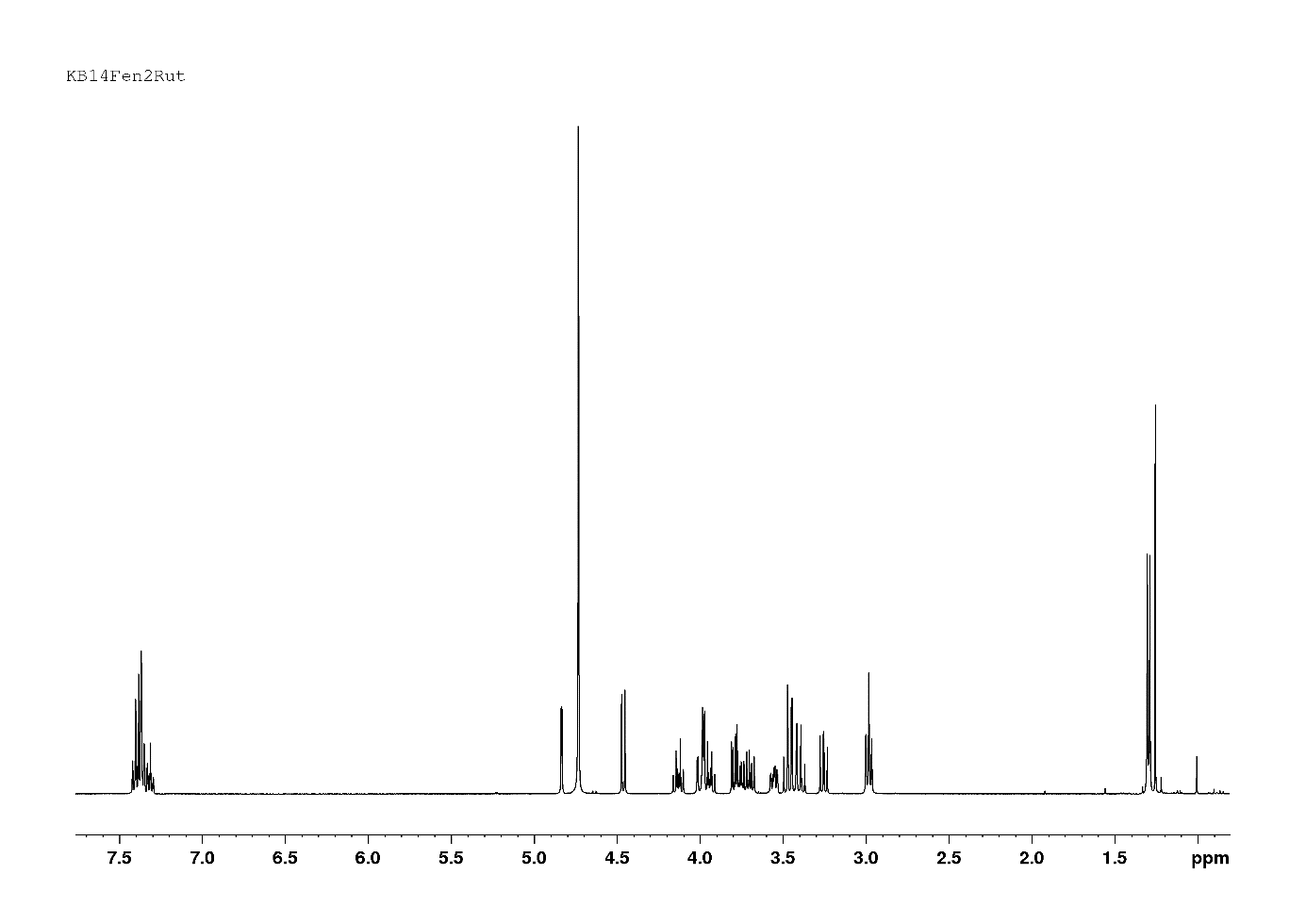


**Fig. S20** ^1^H NMR spectrum of compound **6** (399.87 MHz; D_2_O; 30 ^o^C)


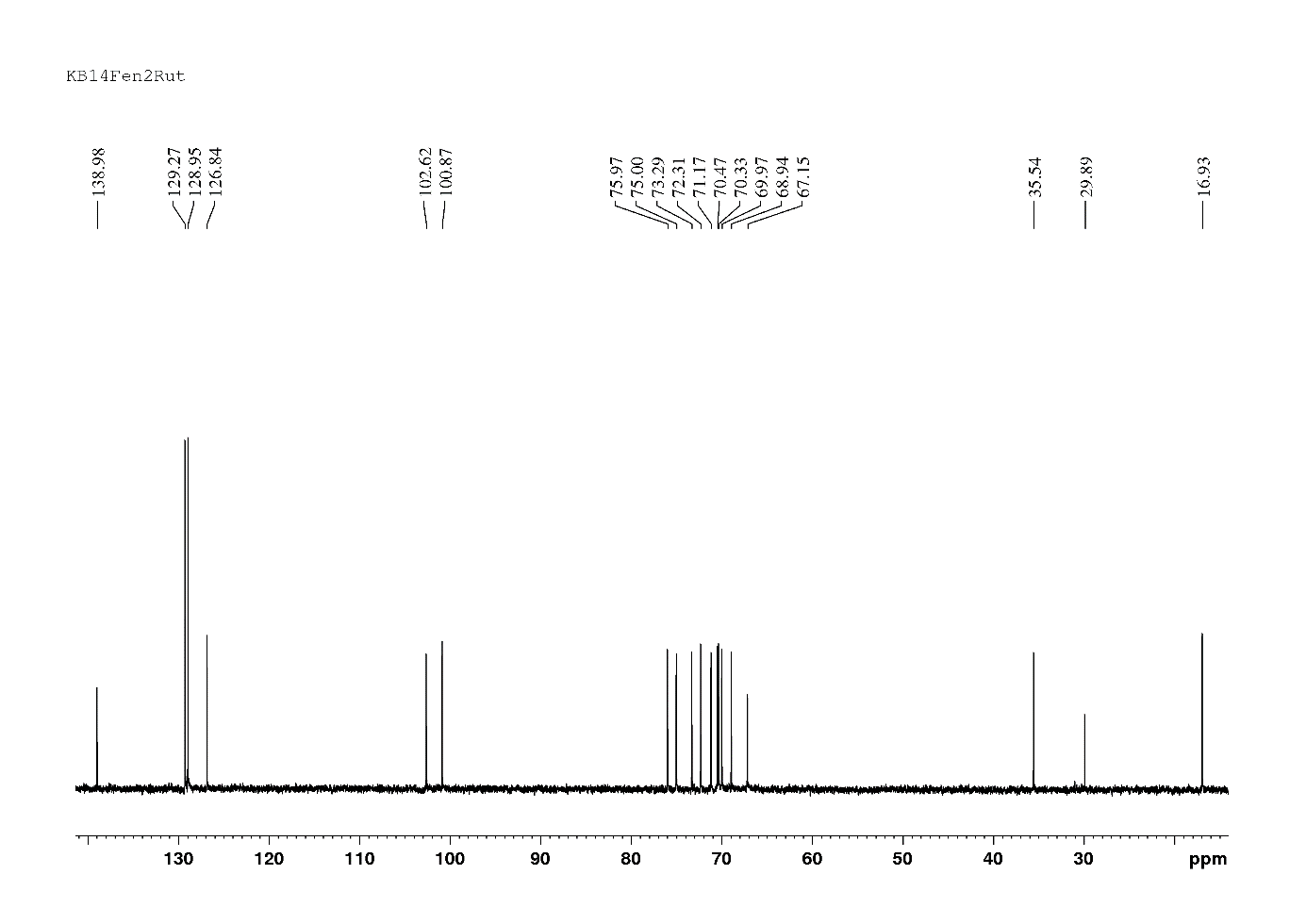


**Fig. S21** ^13^C NMR spectrum of compound **6** (100.55 MHz; D_2_O; 30 ^o^C)


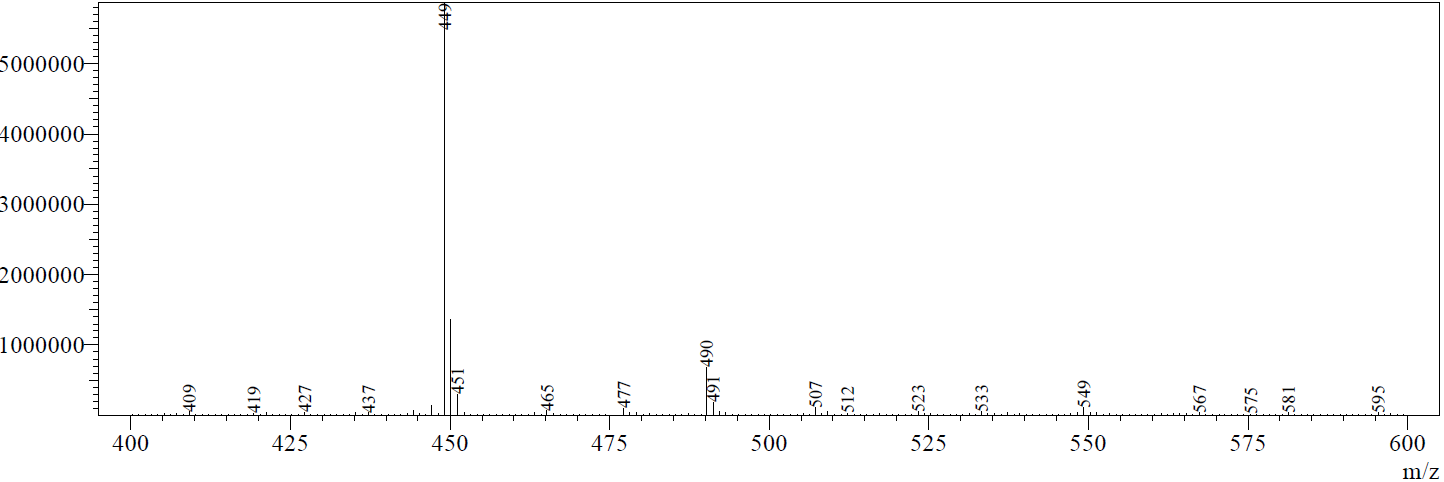


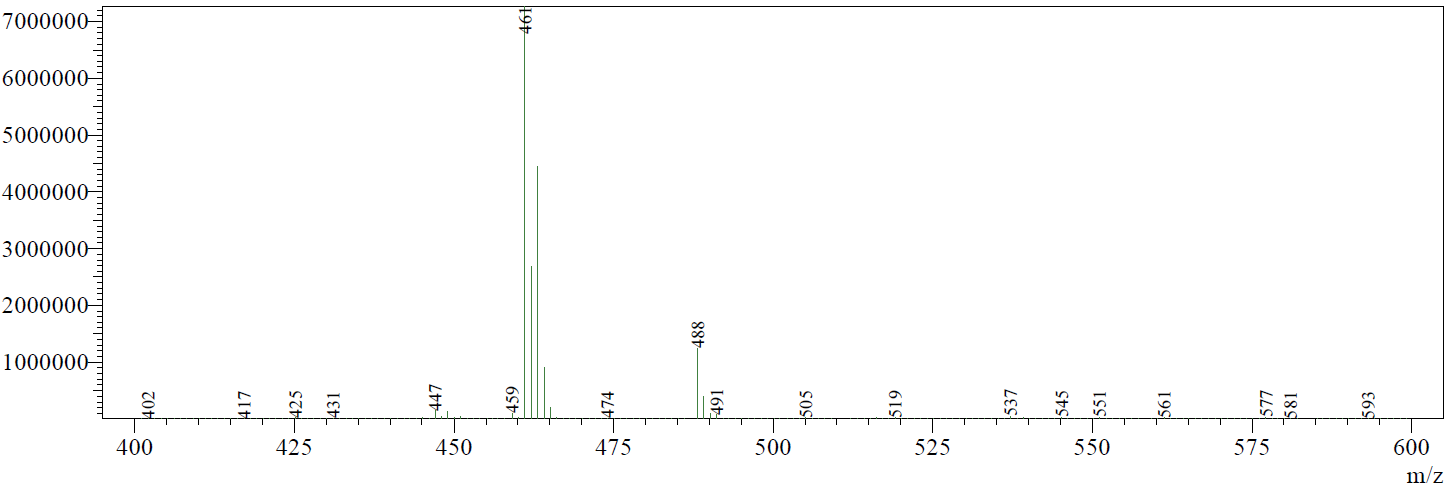


**Fig. S22** Mass spectrometry analysis of a mixture of compounds **7** and **8** generated in a transglycosylation reaction with **2** as a glycosyl donor, 1,2-hexanediol as an acceptor and *Pc*Glc as a catalyst. The preparative transglycosylation reaction was performed as described in the Materials and methods section. Upper MS spectrum (ESI^+^): *m*/*z* = 449 [M+Na]^+^, calculated mass for C_18_H_34_O_11_Na: 449.4. Lower MS spectrum (ESI^–^): *m*/*z* = 461 [M+Cl]^–^, calculated mass for C_18_H_33_O_11_Cl: 460.9. Mass spectra were recorded with a Shimadzu Prominence LC analytical system (Shimadzu, Japan) consisting of a Shimadzu CBM-20A system controller, Shimadzu LC-20AD binary HPLC pump, a Shimadzu CTO-10AS column oven, a Shimadzu SIL-20ACHT cooling autosampler and a Shimadzu SPD-20MA diode array detector. The samples were dissolved in acetonitrile. The mobile phase was composed of acetonitrile; the flow rate was 0.3 mL min^–1^ (25 °C); the injection volume was 1 μL. The MS-ESI parameters were as follows: positive and negative mode; ESI interface voltage: 4.5 kV, –3.5 kV; detector voltage: 1.15 kV; nebulizing gas flow: 1.5 mL min^–1^; drying gas flow: 15 mL min^–1^; heat block temperature: 200 °C; temperature of desolvation line pipe: 250 °C; SCAN mode: 300–600 *m*/*z*. The spectra were analyzed using the software LabSolutions (ver. 5.75 SP2; Shimadzu, Japan)

**
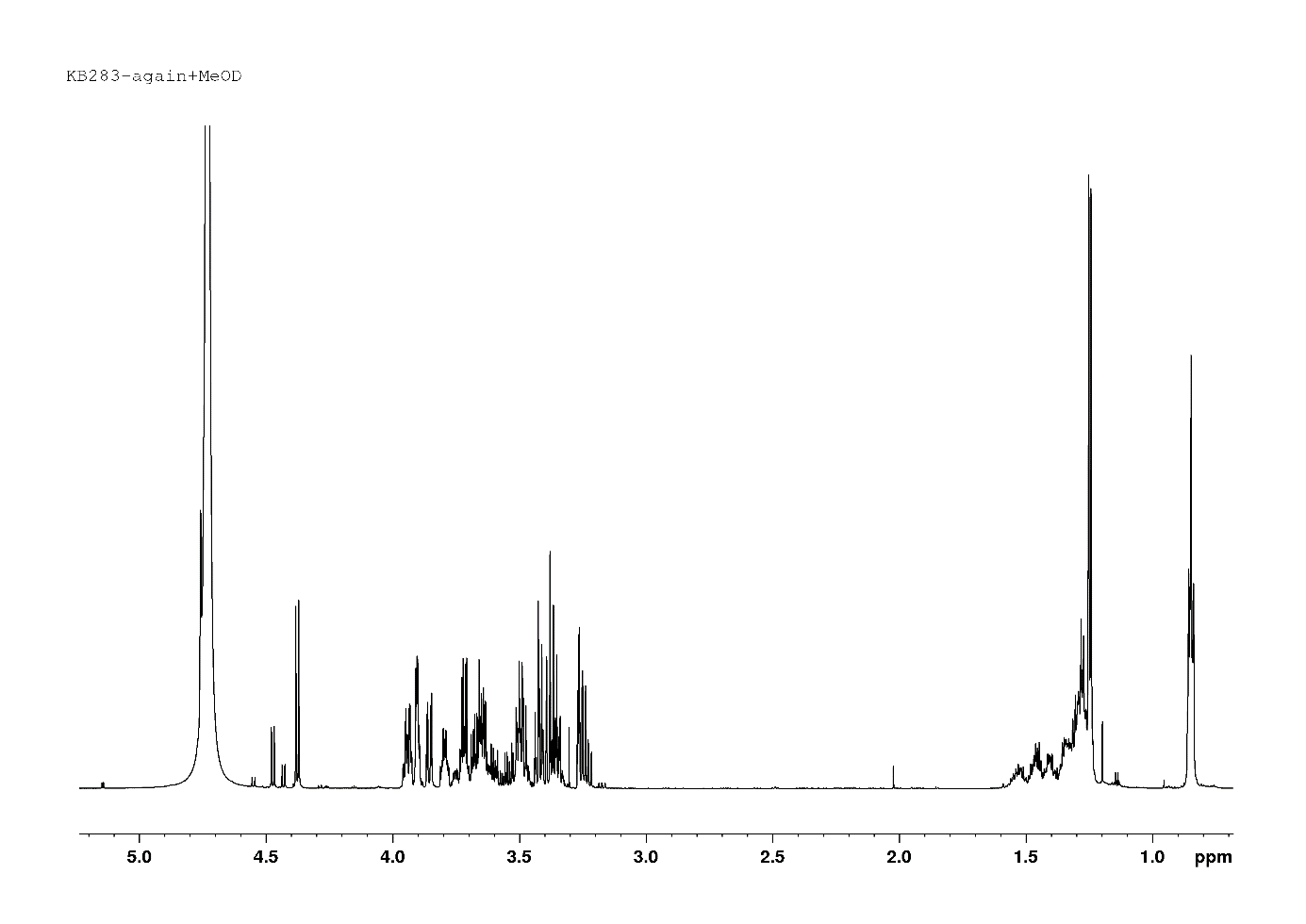
**

**
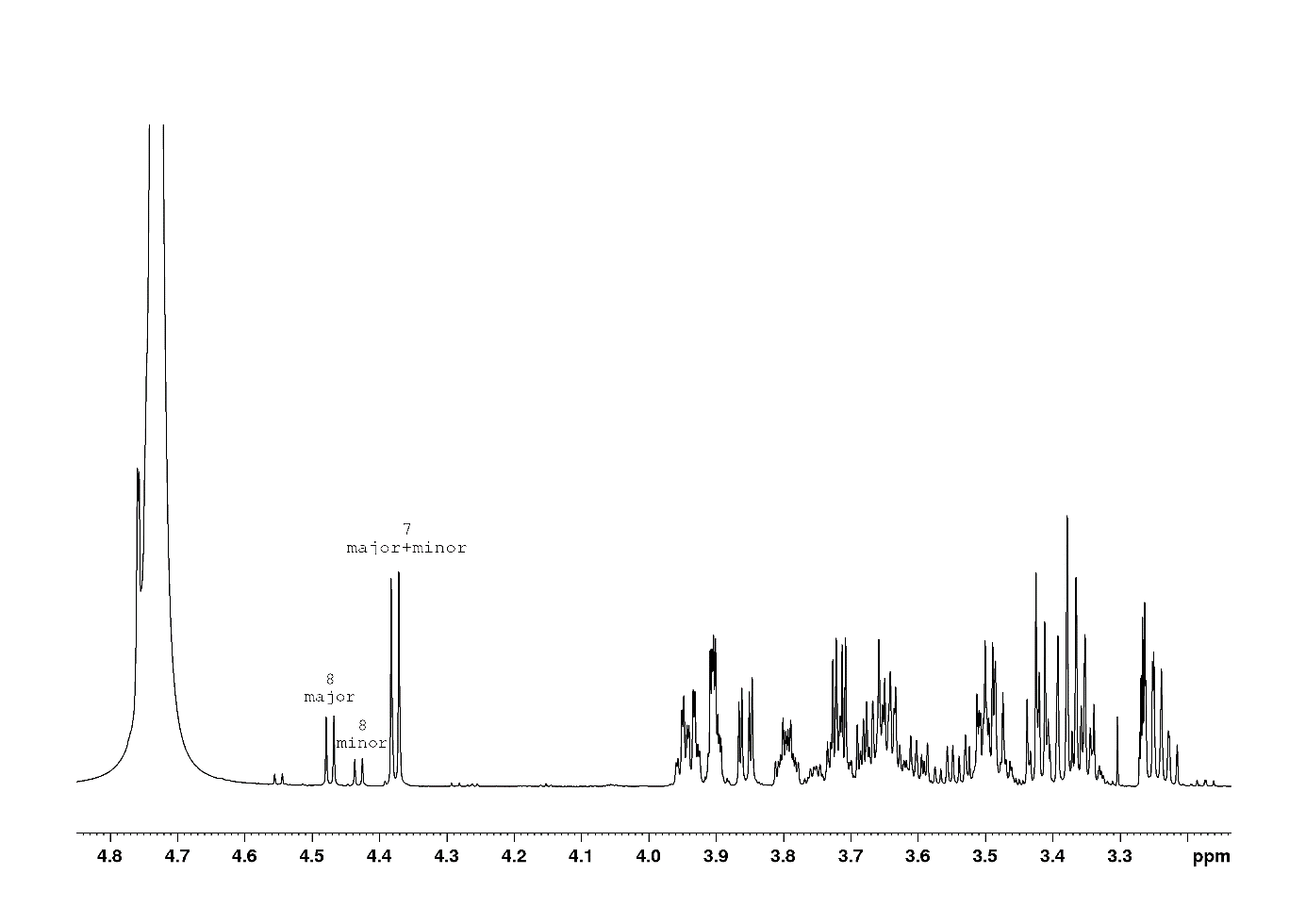
**

**Fig. S23** ^1^H NMR spectrum of both diastereomers of compounds **7** and **8** as a mixture (700.13 MHz; D_2_O and MeOD (9:1, v/v); 30 ^o^C) – overview and close-up


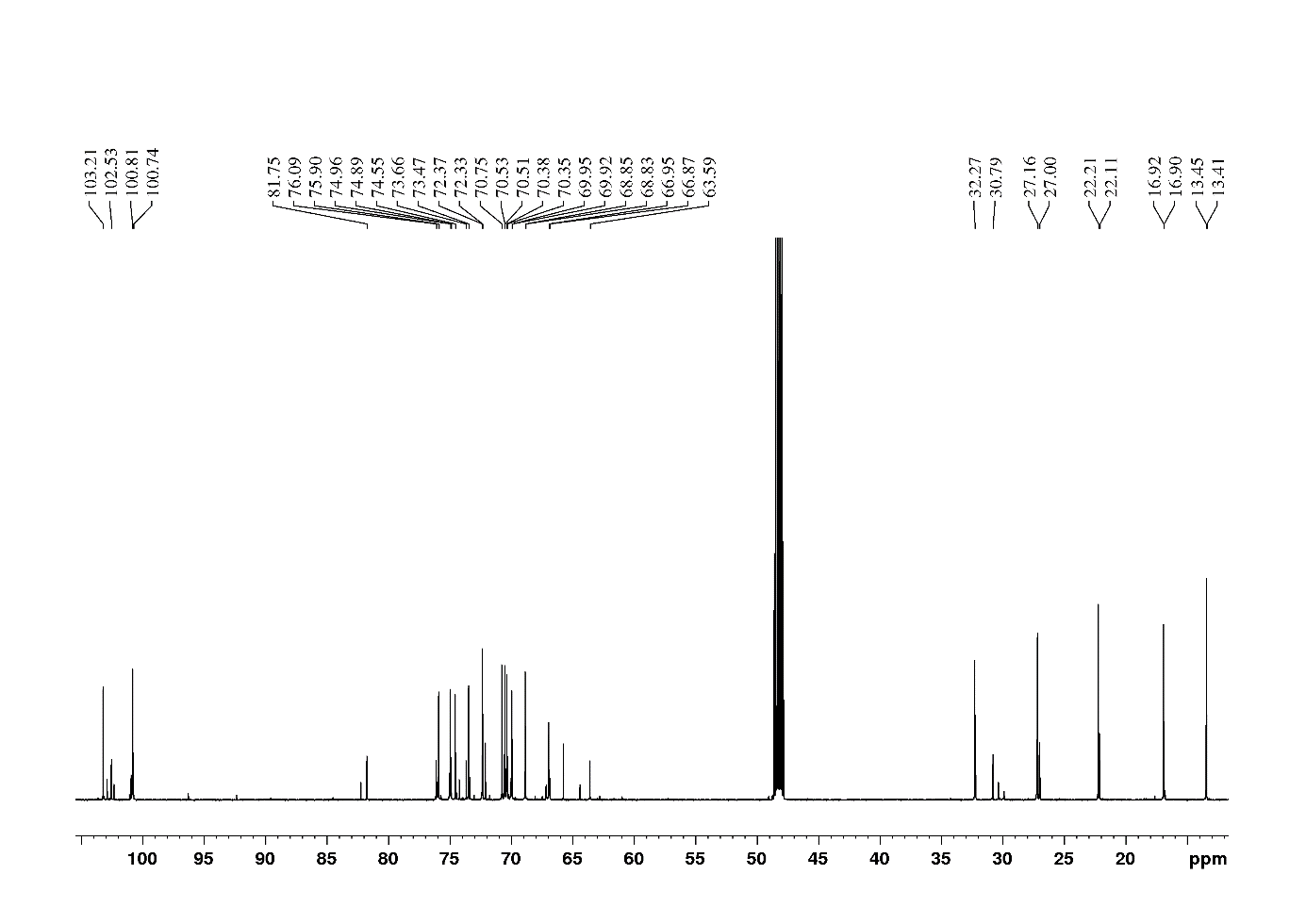


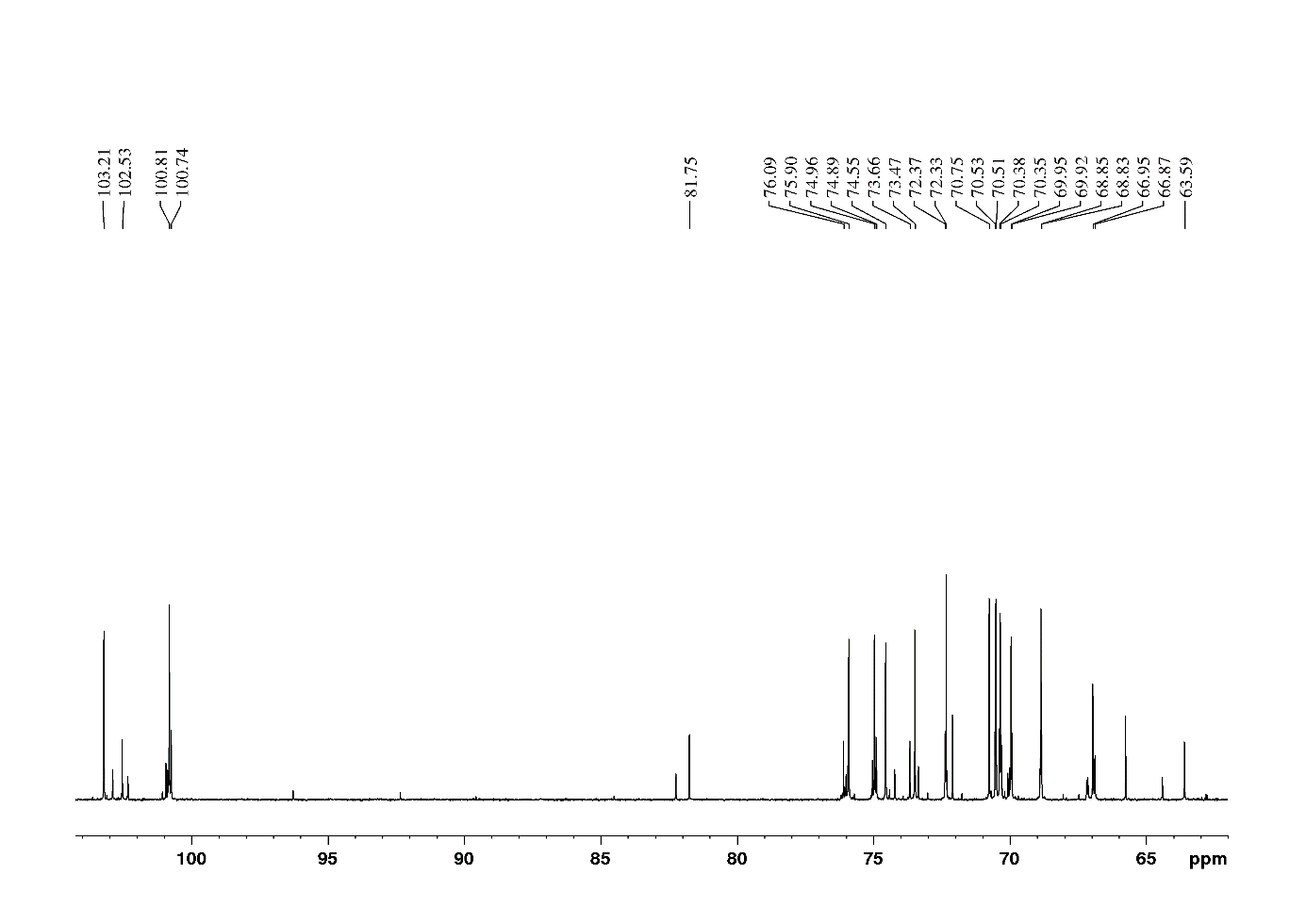


**Fig. S24** ^13^C NMR spectra of both diastereomers of compounds **7** and **8** as a mixture (176.05 MHz; D_2_O and MeOD (9:1, v/v); 30 ^o^C) – overview and close-up. Only signals of major diastereomers, reported in Tables S8 and S9, are labelled

**Fig. S25** Correlation between the conversion of **2**, the formation of transglycosylation product **6** and the acceptor (2-phenylethanol) concentration using *Mc*Glc as a catalyst. The reactions were performed in triplicate in McIlvaine buffer (pH 4.5) at 35 °C with 500 µg mL^–1^ of *Mc*Glc. Thirty minutes after the initiation of the reaction, the amount of **6** (■) and quercetin (○) were determined. The degree of conversion is based on the initial amount of **2** in the reaction mixture (0.3 M)

**a**

**b**

**Fig. S26** Correlation between the conversion of **2**, the formation of transglycosylation product **6** and the acceptor (2-phenylethanol) concentration using *Pc*Glc as a catalyst. The reactions were performed in triplicate in McIlvaine buffer (pH 5.0) at 35 °C with 215 µg mL^–1^ (**a**) or 750 µg mL^–1^ of *Pc*Glc (**b**). Thirty minutes after the initiation of the reaction, the amount of **6** (■) and quercetin (○) were determined. The degree of conversion is based on the initial amount of **2** in the reaction mixture (0.3 M)

**a**

**b**

**Fig. S27** Time courses of conversion of **2** and product formation in transglycosylation reactions with 2-phenylethanol as an acceptor. The formation of quercetin (○) and **6** (□) is shown. *Mc*Glc (A) and *Pc*Glc as catalysts. The reactions were performed at 35 °C with 0.3 M rutin and 1.5 M 2-phenylethanol in McIlvaine buffer (pH 4.5) with 254 µg mL^–1^ of *Mc*Glc (**a**) or in the presence of 0.3 M rutin and 1.2 M 2-phenylethanol in McIlvaine buffer (pH 5.0) with 234 µg mL^–1^ of *Pc*Glc (**b**)

**Fig. S28** Reaction scheme of retaining *Mc*Glc and *Pc*Glc, exhibiting both hydrolysis and transglycosylation activities in the presence of **2**. The first step of the double-displacement mechanism is the formation of a Michaelis complex, followed by the release of the leaving group quercetin, which results in the formation of a covalent glycosyl-enzyme intermediate (rutinosyl-E). The deglycosylation of the enzyme intermediate is initiated by the action of either an activated water molecule or another suitable acceptor molecule such as 2-phenylethanol. In the former case, hydrolysis occurs with the release of rutinose, in the latter case transglycosylation results in the formation of a new rutinoside. If the formation of the glycosyl-enzyme intermediate was rate-limiting, no increase in the overall activity would be observed in the presence of an acceptor because the acceptor would react after the rate-determining step. On the other hand, if the hydrolysis of the intermediate was rate-limiting, the added acceptor would increase the total reaction rate (Fig. 3), which is what was observed for both donors, **1** and **2**

**References**

Benkert P, Biasini M, Schwede T (2011) Toward the estimation of the absolute quality of individual protein structure models. Bioinformatics 27:343–350

Brodsky K, Kutý M, Pelantová H, Cvačka J, Rebroš M, Kotik M, Kutá Smatanová I, Křen V, Bojarová P (2020) Dual substrate specificity of the rutinosidase from *Aspergillus niger* and the role of its substrate tunnel. Int J Mol Sci 21:5671

Pachl P, Kapešová J, Brynda J, Biedermannová L, Pelantová H, Bojarová P, Křen V, Řezáčová P, Kotik M (2020) Rutinosidase from *Aspergillus niger*: crystal structure and insight into the enzymatic activity. FEBS J 287:3315–3327
